# Effects of Maternal Use of the Continuum of Care on Complementary Feeding Practices in Bangladesh: Cross-Sectional Study

**DOI:** 10.2196/76666

**Published:** 2025-10-07

**Authors:** M A Rifat, Rokibul Islam, Rinath Bintey Didar, Joya Bhowmick, Plabon Sarkar, Md Ruhul Amin, Sanjib Saha

**Affiliations:** 1 BRAC Institute of Educational Development, BRAC University, Bangladesh; 2 Institute of Nutrition and Food Science, University of Dhaka, Bangladesh; 3 Department of Food and Nutrition, National College of Home Economics, Dhaka, Bangladesh; 4 Lund University, BMC, Sölvegatan 19, Lund, 223 62, Sweden, 46 736947976

**Keywords:** antenatal care, ANC, postnatal care, PNC, complementary feeding, dietary diversity, minimum acceptable diet, Bangladesh, Bangladesh Demographic and Health Survey, BDHS

## Abstract

**Background:**

The continuum of care (CoC) for maternal health, which includes ≥4 antenatal care (ANC) visits, delivery assisted by skilled birth attendants, and a postnatal care (PNC) visit within 48 hours of delivery, is a crucial health care package associated with survival and improved health outcomes for children and mothers. In addition, the CoC serves as a platform for delivering messages and counseling on child feeding practices. However, the effect of maternal use of the CoC on complementary feeding practices in Bangladesh remains unexamined.

**Objective:**

This study aimed to estimate the effect of maternal use of CoC on complementary feeding practices among children aged 6 to 23 months in Bangladesh.

**Methods:**

Data from 2 consecutive nationally representative surveys—the Bangladesh Demographic and Health Survey 2017-2018 and 2022—were analyzed. Observations corresponding to the CoC for maternal health and complementary feeding indicators, including (1) timely introduction of solid, semisolid, and soft food (ISSSF); (2) minimum meal frequency (MMF); (3) minimum dietary diversity (MDD); and (4) minimum acceptable diet (MAD), were merged to prepare the analyzed samples. The differences in complementary feeding practice indicators by maternal use of the CoC were observed using chi-square tests. Multivariable logistic regression models were used to observe the associations.

**Results:**

The analysis included 887, 4967, 4967, and 4967 mother-child pairs for the timely ISSSF, MMF, MDD, and MAD indicators, respectively. The status of complementary feeding indicators was significantly different (*P*<.05) by maternal use of the full CoC, ≥4 ANC visits, status of receiving PNC within 48 hours of birth, maternal educational level, husband’s educational level, maternal occupation, wealth index of families, maternal perceived problems with accessing health care, and division of residence. Mothers who received the full CoC were 29% and 32% more likely to meet the MDD (adjusted odds ratio [AOR] 1.29, 95% CI 1.10-1.51; *P*=.002) and MAD (AOR 1.32, 95% CI 1.13-1.55; *P*=.001) than those who did not receive the full CoC, respectively. Among the individual components of the CoC, mothers who received ≥4 ANC visits were 23%, 31%, and 34% more likely to meet the MMF (AOR 1.23, 95% CI 1.05-1.45; *P*=.01), MDD (AOR 1.31, 95% CI 1.13-1.51; *P*<.001), and MAD (AOR 1.34, 95% CI 1.15-1.56; *P*<.001) than those with fewer ANC visits, respectively. Furthermore, the effects of delivery assisted by skilled birth attendants and receiving PNC within 48 hours of delivery on complementary feeding indicators were also statistically insignificant (*P*≥.05).

**Conclusions:**

Maternal use of the CoC appears to be effective in improving MDD and MAD among children aged 6 to 23 months in Bangladesh. The findings highlight the pivotal role of recommended ANC visits in promoting complementary feeding practices and further suggest opportunities to amplify the impact of CoC on timely ISSSF and MMF.

## Introduction

### Background

The age of 6 to 23 months constitutes the largest proportion of the first 1000 days of life, which are referred to as the critical window of opportunity [1]. This age group is characterized by rapid body growth, cognitive development, language acquisition, personality development, and outstanding learning skills [1,2]. Therefore, children aged 6 to 23 months critically require proper nutrition. This is primarily met through appropriate complementary feeding, which demonstrates a significant association with childhood growth [3,4]. As infants grow, their requirement for essential micronutrients increases, which is not fulfilled by breast milk alone, requiring quality and frequency of complementary food. Evidence shows that proper complementary feeding can help maintain adequate supply of essential macro- and micronutrients (eg, protein, calories, vitamin A, iron, and zinc), which are essential for linear growth, cognitive development, and protecting young children from deficiency disorders [5-7]. Furthermore, poor complementary feeding results in growth faltering and frequent infection in the short term, whereas the long-term consequences include an elevated risk of diabetes, obesity, and poor neuromuscular development [8,9]. Children aged 6 to 23 months are highly vulnerable to malnutrition; therefore, poor complementary feeding can result in growth faltering, which might be irreversible in later life [10]. Apart from undernutrition, the period of complementary feeding is a crucial opportunity to prevent overweight and obesity during adulthood [11].

Initiation of complementary feeding at the age of 6 months triggers the transition from breastfeeding to family foods; however, the process should take several factors into careful consideration, including children’s growth patterns, requirements of essential nutrients, appetite, satiety, and quality of food [12]. The frequency, quantity, and quality of complementary food differ by children’s age, so the provision of age-appropriate complementary feeding requires caregivers skilled or educated in complementary feeding practices [13]. One of the most considered strategies to improve caregivers’ complementary feeding practices is to provide them with knowledge and motivation, especially through interventions during the antenatal and postnatal periods, which are often integrated with antenatal care (ANC) and postnatal care (PNC) [14].

The continuum of care (CoC) for maternal health offers a package of health care services from pregnancy to the postnatal period that are essential for the survival and well-being of the mother and newborn. These services include at least 4 ANC visits by skilled care providers during pregnancy, delivery assisted by skilled birth attendants (SBAs), and a PNC visit within 48 hours of delivery. These services provide pregnant mothers with opportunities to be in contact with trained health care providers during critical periods. Therefore, ANC and PNC are platforms to deliver nutrition education and sensitize mothers on childcare and complementary feeding. Furthermore, delivery by SBAs indicates mothers’ positive health care–seeking behavior, which may be carried forward to the practice of recommended complementary feeding. Evidence shows that interventions to improve complementary feeding practice target pregnant mothers, considering ANC as a valuable platform to provide counseling and education [15-17]. In low- and middle-income countries, nutrition education and complementary feeding interventions appeared to exhibit significant positive results on linear growth among children [4,18].

### Objectives

Despite invaluable benefits, complementary feeding practices in Bangladesh demonstrate a frustrating picture, with only 20% of children aged 6 to 23 months receiving a minimum acceptable diet (MAD) in 2024 [19,20]. According to the latest Bangladesh Demographic and Health Survey (BDHS) in 2022, only 30% of children were fed with an MAD, and this unfortunately experienced a drop from 35% in 2017 [21,22]. Addressing malnutrition and micronutrient deficiencies through the promotion of complementary feeding practices is one of the key priorities in the second national plan of action of nutrition in Bangladesh [23]. One of the strategies to achieve nutrition goals is mainstreaming nutrition, where many nutrition services are integrated into health services such as ANC and PNC, with the hypothesis that mothers who undergo maternal health care services will exhibit better performance in child feeding practices [23,24]. However, no previous study has investigated the effect of maternal use of the CoC on complementary feeding practices in Bangladesh. Using 2 nationally representative surveys, this study aimed to fill this research gap through estimating the overall effect of maternal use of the CoC and its components on complementary feeding practices over the survey periods. The findings might help policymakers understand how the benefits of maternal use of CoC translate to complementary feeding practices, identifying priorities and ways forward.

## Methods

### Study Design and Samples

In this study, the STROBE (Strengthening the Reporting of Observational Studies in Epidemiology) checklist was used as the reporting guideline [25]. The STROBE checklist is provided in Checklist 1. We used 2 nationally representative cross-sectional surveys as data sources. These sources were the BDHS of 2017-2018 and 2022. The surveys applied 2-stage stratified cluster random sampling and obtained a response rate of more than 98%. In both surveys, the samples represented mothers of children aged 6 to 23 months in Bangladesh. The sample represented both urban and rural mothers. Families were recruited for the survey based on a random selection process and subject to their consent to participate. The interviews were conducted at the households of selected families. The detailed methods are outlined elsewhere [21,22].

However, in brief, the study only analyzed samples with complete information about maternal use of the CoC and coupled with the respective complementary feeding indicators (ie, introduction to solid, semisolid, and soft food [ISSSF]; minimum meal frequency [MMF]; minimum dietary diversity [MDD]; and MAD). Therefore, women who were interviewed for birth history information (ie, data about women who had given birth) were filtered from the dataset for inclusion in this study. Women who had a live birth within the last 3 and 2 years before the surveys, respectively, for the BDHS 2017-2018 and 2022 were then retrieved. These retrieved observations served as the source datasets to estimate maternal use of the CoC or the exposure variables. Finally, the maternal CoC variables were merged with variables accommodating the respective complementary feeding indicators, making up the analyzed samples for each complementary feeding indicator.

### Exposure Variables

The exposure variables were maternal use of the CoC and its 3 components. The components included (1) at least 4 ANC visits by skilled health care providers during pregnancy, (2) SBA-assisted delivery, and (3) at least one PNC visit by skilled health care providers within 48 hours of delivery. In this study, skilled health care providers were denoted as physicians, nurses, midwives or paramedics, family welfare visitors, community SBAs, and subassistant community medical officers. Mothers were coded as 1 if they received all 3 components of the CoC and as 0 otherwise. Similarly, mothers who used any individual component of the CoC were coded as 1 or as 0 otherwise and analyzed separately.

### Outcome Variables

The outcome variables included complementary feeding indicators (eg, ISSSF, MMF, MDD, and MAD). The status of ISSSF was estimated by calculating the proportion of children aged 6 to 8 months who were fed solid, semisolid, or soft food during the previous day or night. MMF refers to the minimum number of meals provided among children aged 6 to 23 months by their age category (6-8 months and 9-23 months) and breastfeeding status (breastfed and nonbreastfed). Furthermore, MDD indicates the minimum number of food groups fed to children aged 6 to 23 months. Finally, the estimation of the MAD considers both the MMF and MDD, indicating that a child had an MAD if both the MMF and MDD were met. Calculation of the outcome variables was based on BDHS guidelines [26]. Positive status of ISSSF, MMF, MDD, and MAD was coded as 1 and as 0 otherwise.

### Covariates

Covariates were selected based on their expected association with the exposure variables and evidence from the published literature [27-29]. Therefore, the covariates considered were age at delivery (<19 years, 19-30 years, and 31-49 years); educational level (no education, primary education, secondary education, and higher education); husband’s educational level (no education, primary education, secondary education, and higher education); occupation (not working or working); husband’s occupation (not working or working); parity (1, 2-3, and 3); whether they had ever terminated a pregnancy (never or no and ever or yes); desired pregnancy (yes or no); wealth index (poorest, poorer, middle, richer, or richest); any exposure to television, radio, or the news (yes or no); whether they were facing problems such as obtaining permission and money and long distance to access health care (big problem or not a big problem); religion (Muslim or other); place of residence (rural or urban); division of residence (Barisal, Chittagong, Dhaka, Khulna, Mymensingh, Rajshahi, Rangpur, or Sylhet), and survey round (BDHS 2017-2018 or 2022).

### Statistical Analysis

Cross-tabulation was used to observe the distribution of complementary feeding status by maternal use of the CoC and covariates, and the differences were observed using chi-square tests. The binary nature of the outcome variables led to the consideration of univariable and multivariable logistic regression models to estimate the effect of maternal use of the CoC, further divided into its individual components, on complementary feeding practices among children aged 6 to 23 months. Therefore, the individual effect of maternal use of the full CoC, receiving ≥4 ANC visits, delivery assisted by SBAs, and receiving PNC within 48 hours of delivery on the 4 complementary feeding indicators was observed, resulting in a total of 16 multivariable logistic regression models to observe the associations. The logistic regression models were adjusted for sampling weight, strata, primary sampling unit, and covariates using the *svy* command. Multicollinearity was observed using the SE of the odds ratio and variance inflation factor (VIF), considering an SE of >2 or VIF of >5 as indicators of the presence of multicollinearity [30,31]. All the analyses were conducted considering *P*<.05 as statistically significant. The Stata software (version 17; StataCorp) was used to conduct the statistical analyses.

### Ethical Considerations

This study was exempted from receiving approval from an ethical review committee because it was conducted based on publicly available BDHS data, which involved interviewing mothers of children under 2 years of age to collect information. However, the BDHS was approved by the ICF Macro Institutional Review Board (United States) following the overall requirements of Title 45, Part 46 of the Code of Federal Regulations, “Protection of Human Subjects.” Moreover, as the data collection procedure was carried out in Bangladesh, the National Research Ethics Committee of the Bangladesh Medical Research Council, Dhaka, Bangladesh, also reviewed and approved the BDHS. Before enrollment in the study, informed consent was obtained verbally from each participant and their intimate partners (for all ever-married women aged 15‐49 years). Verbal consent was considered the most suitable option to ensure the participation of respondents who were incapable of reading. Very young children who were under 5 years of age and were born in 2014 or later were also considered in the sample when the data collection procedure was conducted by the BDHS. Verbal consent for children’s participation in the survey was obtained from their mothers, who acted as legal guardians and provided authorization on behalf of their children. Before using the data for research purposes, the responses were deidentified. No compensation was provided to any participants for their responses or the information used in this research. Furthermore, this paper and its supplementary material do not contain any identifiable information or images of participants whose responses were used to conduct this study. Detailed ethical aspects and consent forms are available in the BDHS website and report.

## Results

**Figure 1.**
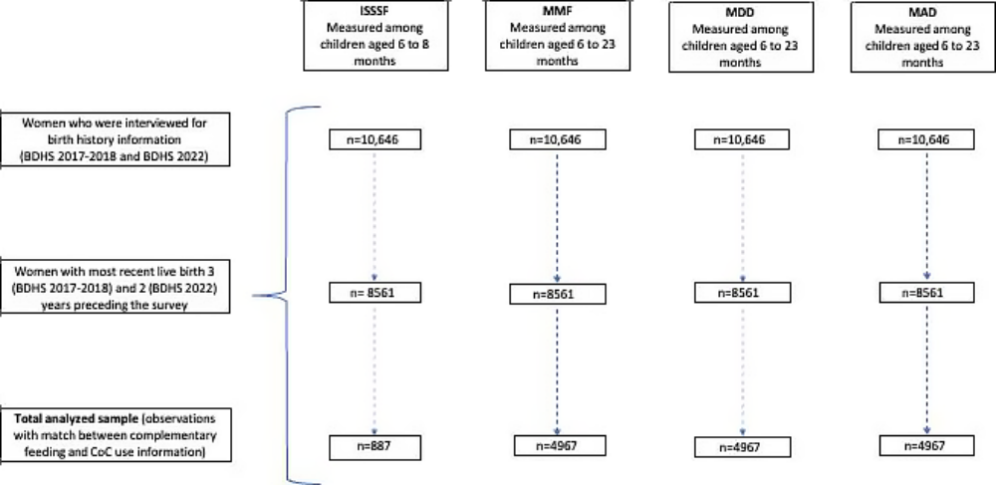
Selection process of the analyzed sample from the Bangladesh Demographic and Health Survey (BDHS) 2017-2018 and 2022 data. CoC: continuum of care; ISSSF: introduction to solid, semisolid, and soft food; MAD: minimum acceptable diet; MDD: minimum dietary diversity; MMF: minimum meal frequency.

**Figure 2.**
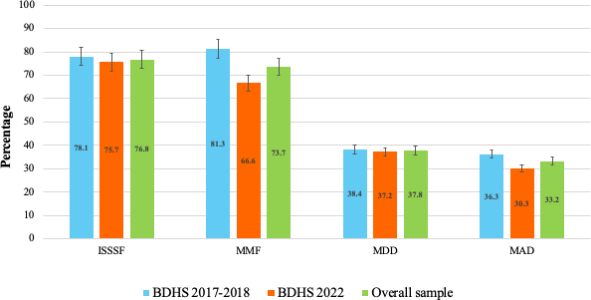
Status of complementary feeding practices in Bangladesh calculated from the analyzed sample. BDHS: Bangladesh Demographic and Health Survey; ISSSF: introduction to solid, semisolid, and soft food; MAD: minimum acceptable diet; MDD: minimum dietary diversity; MMF: minimum meal frequency.

Figure 1 shows the results of the sample selection process. A total of 8561 observations were included in the analysis (n=887, 10.36% for ISSSF; n=4967, 58.02% for MMF; n=4967, 58.02% for MDD; and n=4967, 58.02% for MAD). The status of timely ISSSF, MMF, MDD, and MAD is shown in Figure 2.

The distribution of outcome variables by maternal use of the CoC, ANC visits, delivery assisted by SBAs, PNC, and maternal socioeconomic characteristics is shown in Table 1. The analysis shows that the distributions of all 4 outcome variables were statistically significantly (*P*<.05) and differed by maternal use of the CoC, status of receiving ≥4 ANC visits, status of receiving PNC within 48 hours of birth, maternal educational level, husband’s educational level, maternal occupation, wealth index, perceived problems with accessing health care, and division of residence. In addition, the status of MMF, MDD, and MAD significantly differed by maternal status of media exposure and place of residence, whereas delivery assisted by SBAs, husband’s occupation, and parity were variables that showed significant differences in the distribution of MDD and MAD.

**Table 1.** Distribution of complementary feeding indicators by status of maternal use of the continuum of care (CoC) and background characteristics^a^.

Independent variable	ISSSF^b^, n (%)			MMF^c^, n (%)			MDD^d^, n (%)			MAD^e^, n (%)		
	No	Yes	*P* value	No	Yes	*P* value	No	Yes	*P* value	No	Yes	*P* value
CoC use			.001			<.001			<.001			<.001
Yes	39 (13.93)	241 (86.07)		290 (19.42)	1203 (80.58)		746 (49.97)	747 (50.03)		811 (54.32)	682 (45.6)	
≥4 ANC^f^ visits			.02			<.001			<.001			<.001
Yes	70 (17.16)	338 (82.84)		450 (20.39)	1757 (79.61)		1173 (53.15)	1034 (46.85)		1277 (57.86)	930 (42.14)	
Delivery by SBAs^g^			.08			.11			<.001			<.001
Yes	108 (18.95)	462 (81.05)		764 (24.93)	2300 (75.07)		1763 (57.54)	1301 (42.46)		1926 (62.32)	1138 (37.14)	
PNC^h^ within 48 hours of birth			.003			<.001			<.001			<.001
Yes	85 (17.10)	412 (82.90)		602 (22.82)	2036 (77.18)		1504 (57.01)	1134 (42.99)		1619 (61.37)	1019 (38.63)	
Age at delivery (y)			.39			.09			.74			.47
<19	20 (17.70)	93 (82.30)		177 (24.05)	559 (75.95)		448 (60.87)	288 (39.13)		482 (65.49)	254 (34.51)	
19-30	138 (21.94)	491 (78.06)		917 (26.60)	2531 (73.40)		2140 (62.06)	1308 (37.94)		2305 (66.84)	1143 (33.15)	
31-49	26 (17.93)	119 (82.07)		183 (23.37)	600 (76.63)		477 (60.92)	306 (39.08)		507 (64.75)	276 (35.25)	
Educational level			.03			<.001			<.001			<.001
No education	12 (26.09)	34 (73.91)		90 (32.85)	184 (67.15)		217 (79.20)	57 (20.80)		227 (82.85)	47 (17.15)	
Primary education	48 (23.41)	157 (76.59)		338 (27.06)	911 (72.94)		903 (72.30)	346 (27.70)		936 (74.94)	313 (25.06)	
Secondary education	104 (21.89)	371 (78.11)		659 (26.31)	1846 (73.69)		1528 (61.00)	977 (39.00)		1657 (66.15)	848 (33.85)	
Higher education	20 (12.42)	141 (87.58)		190 (20.23)	749 (79.77)		417 (44.41)	522 (55.59)		474 (50.48)	465 (49.52)	
Husband’s educational level			.047			<.001			<.001			<.001
No education	30 (22.56)	103 (77.44)		230 (32.72)	473 (67.28)		523 (74.40)	180 (25.60)		553 (78.66)	150 (21.34)	
Primary education	66 (23.66)	213 (76.34)		412 (25.99)	1173 (74.01)		1070 (67.51)	515 (32.49)		1116 (70.41)	469 (29.59)	
Secondary education	66 (21.57)	240 (78.43)		426 (26.30)	1194 (73.70)		989 (61.05)	631 (38.95)		1081 (66.73)	539 (33.27)	
Higher education	21 (12.88)	142 (87.12)		189 (18.94)	809 (81.06)		444 (44.49)	554 (55.51)		500 (50.10)	498 (49.90)	
Occupation status			.007			<.001			.02			<.001
Not working	144 (23.19)	477 (76.81)		978 (28.83)	2414 (71.17)		2130 (62.79)	1262 (37.21)		2310 (68.10)	1082 (31.90)	
Working	40 (15.09)	225 (84.91)		299 (19.00)	1275 (81.00)		934 (59.34)	640 (40.66)		983 (62.45)	591 (37.55)	
Husband’s occupation status			.78			.13			.04			.02
Not working	4 (23.53)	13 (76.47)		32 (32.32)	67 (67.68)		71 (71.72)	28 (28.28)		77 (77.78)	22 (22.22)	
Working	180 (20.71)	689 (79.29)		1236 (25.52)	3608 (74.48)		2980 (61.52)	1864 (38.48)		3200 (66,06)	1644 (33.94)	
Parity			.13			.07			<.001			<.001
1	57 (17.59)	267 (82.41)		445 (23.87)	1419 (76.13)		1067 (57.24)	797 (42.76)		1172 (62.88)	692 (37.12)	
2-3	101 (21.77)	363 (78.23)		694 (26.91)	1885 (73.09)		1628 (63.13)	951 (36.87)		1740 (67.7)	839 (32.53)	
>3	26 (26.26)	73 (73.74)		138 (26.34)	386 (73.66)		370 (70.61)	154 (29.39)		382 (72.90)	142 (27.10)	
Ever terminated a pregnancy			.52			.16			.23			.21
Yes	30 (18.87)	129 (81.13)		208 (23.83)	665 (76.17)		523 (59.91)	350 (40.09)		563 (64.49)	310 (35.51)	
Desired pregnancy			.65			.24			.78			.67
No	41 (21.93)	146 (78.07)		251 (24.27)	783 (75.73)		642 (62.09)	392 (37.91)		680 (65.76)	354 (34.24)	
Wealth index			.03			<.001			<.001			<.001
Poorest	51 (27.27)	136 (72.73)		328 (30.14)	737 (69.86)		780 (73.93)	275 (26.07)		817 (77.44)	238 (22.56)	
Poorer	36 (20.22)	142 (79.78)		285 (27.51)	751 (72.49)		684 (66.02)	352 (33.98)		726 (70.08)	310 (29.92)	
Middle	31 (16.85)	153 (83.15)		235 (25.54)	685 (74.46)		592 (64.35)	328 (35.65)		630 (68.48)	290 (31.52)	
Richer	43 (23.50)	140 (76.50)		244 (24.67)	745 (75.33)		580 (58.65)	409 (41.35)		632 (63.90)	357 (36.10)	
Richest	23 (14.84)	132 (85.16)		195 (20.17)	772 (79.83)		429 (44.36)	538 (55.64)		489 (50.57)	478 (49.43)	
Media exposure^i^			.12			<.001			<.001			<.001
Yes	97 (18.95)	415 (81.05)		657 (21.97)	2334 (78.03)		1674 (55.97)	1317 (44.03)		1807 (60.41)	1184 (39.59)	
Access to health care^j^			.005			<.001			<.001			<.001
Not a big problem	61 (16.27)	314 (83.73)		434 (21.35)	1599 (78.65)		1175 (57.80)	858 (42.20)		1265 (62.22)	768 (37.78)	
Big problem	123 (24.02)	389 (75.98)		843 (28.73)	2091 (71.27)		1890 (64.42)	1044 (35.58)		2029 (69.15)	905 (30.85)	
Religion			.96			.10			.46			.53
Muslim	168 (20.77)	641 (79.23)		1191 (26.00)	3389 (74.00)		2833 (61.86)	1747 (38.14)		3043 (66.44)	1537 (33.56)	
Others	16 (20.51)	62 (79.49)		86 (22.22)	301 (77.78)		232 (59.95)	155 (40.05)		251 (64.86)	136 (35.14)	
Residence			.08			.001			<.001			<.001
Urban	46 (17.10)	223 (82.90)		368 (22.63)	1258 (77.37)		909 (55.90)	717 (44.10)		990 (60.89)	636 (39.11)	
Rural	138 (22.33)	480 (77.67)		909 (27.21)	2432 (72.79)		2156 (64.53)	1185 (35.47)		2304 (68.96)	1037 (31.04)	
Division of residence			<.001			<.001			<.001			<.001
Barisal	20 (19.23)	84 (80.77)		168 (31.82)	360 (68.18)		355 (67.23)	173 (32.77)		375 (71.02)	153 (28.98)	
Chittagong	49 (36.84)	84 (63.16)		292 (35.57)	529 (64.43)		541 (65.90)	280 (34.10)		588 (71.62)	233 (28.38)	
Dhaka	30 (24.00)	95 (76.00)		170 (23.55)	552 (76.45)		418 (57.89)	304 (42.11)		452 (62.60)	270 (37.40)	
Khulna	7 (7.29)	89 (92.71)		79 (15.11)	444 (84.89)		279 (53.35)	244 (46.65)		297 (56.79)	226 (43.21)	
Mymensingh	14 (12.50)	98 (87.50)		137 (22.03)	485 (77.97)		369 (59.32)	253 (40.68)		401 (64.470)	221 (35.53)	
Rajshahi	13 (15.85)	69 (84.15)		124 (24.60)	380 (75.40)		307 (60.91)	197 (39.09)		328 (65.08)	176 (34.92)	
Rangpur	15 (13.76)	94 (86.24)		117 (19.70)	477 (80.30)		338 (56.90)	256 (43.10)		371 (62.46)	223 (37.54)	
Sylhet	36 (28.57)	90 (71.43)		190 (29.10)	463 (70.90)		458 (70.14)	195 (29.86)		482 (73.81)	171 (26.19)	
Survey round			.17			<.001			.37			<.001
BDHS^k^ 2017-2018	77 (18.73)	334 (81.27)		426 (17.63)	1991 (82.37)		1476 (61.07)	941 (38.93)		1526 (63.14)	891 (36.86)	
BDHS 2022	107 (22.48)	369 (77.52)		851 (33.37)	1699 (66.63)		1589 (62.31)	961 (37.69)		1768 (69.33)	782 (30.67)	

a For dichotomous responses (yes or no), only the positive response (yes) is presented.

b ISSSF: introduction to solid, semisolid, and soft food.

c MMF: minimum meal frequency.

d MDD: minimum dietary diversity.

e MAD: minimum acceptable diet.

f ANC: antenatal care.

g SBA: skilled birth attendant.

h PNC: postnatal care.

i Reading newspapers, watching television, or listening to the radio.

j Perception that receiving permission to visit health care facilities, not having money to access health care, or the distance to health care facilities are big problems.

k BDHS: Bangladesh Demographic and Health Survey.

The association between complementary feeding practices and maternal use of the CoC and its components is shown in Table 2. The analysis shows that maternal use of either the CoC or its components had no significant association with timely ISSSF among children aged 6 to 8 months. On the other hand, MDD and MAD among children aged 6 to 23 months were statistically significantly associated with maternal use of the CoC and receiving ≥4 ANC visits.

**Table 2.** Multivariable regression models demonstrating the association between maternal use of the continuum of care (CoC) and complementary feeding indicators.

Exposure variable	ISSSF^a^		MMF^b^		MDD^c^		MAD^d^	
	AOR^e^ (95% CI)	*P* value	AOR^e^ (95% CI)	*P* value	AOR^e^ (95% CI)	*P* value	AOR^e^ (95% CI)	*P* value
Full CoC use								
No	Reference	—^f^	Reference	—	Reference	—	Reference	—
Yes	1.30 (0.77-2.20)	.33	1.13 (0.94-1.37)	.20	1.29^g^ (1.10-1.51)	.002	1.32^g^ (1.13-1.55)	.001
≥4 ANC^h^ visits								
No	Reference	—	Reference	—	Reference	—	Reference	—
Yes	1.00 (0.64-1.57)	.98	1.23^g^ (1.05-1.45)	.01	1.31^g^ (1.13-1.51)	<.001	1.34^g^ (1.15-1.56)	<.001
Delivery by SBAs^i^								
No	Reference	—	Reference	—	Reference	—	Reference	—
Yes	0.98 (0.63-1.52)	.92	0.94 (0.80-1.11)	.49	1.04 (0.89-1.22)	.62	1.03 (0.87-1.21)	.77
PNC^j^ within 48 hours of birth								
No	Reference	—	Reference	—	Reference	—	Reference	—
Yes	1.27 (0.83-1.93)	.27	1.05 (0.89-1.24)	.55	0.99 (0.85-1.15)	.88	1.03 (0.88-1.20)	.72

a ISSSF: introduction to solid, semisolid, and soft food.

b MMF: minimum meal frequency.

c MDD: minimum dietary diversity.

d MAD: minimum acceptable diet.

e AOR: adjusted odds ratio. Adjusted for primary sampling unit, sampling strata, sampling weight, age at last delivery, educational level, husband’s educational level, occupation, husband’s occupation, parity, whether they had ever terminated a pregnancy, desired pregnancy, wealth index, media exposure, access to health care, religion, residence, division of residence, and survey round.

f Not applicable.

g *P*<.05.

h ANC: antenatal care.

i SBA: skilled birth attendant.

j PNC: postnatal care.

Mothers who received the full CoC were 29% and 32% more likely to provide an MDD (adjusted odds ratio [AOR] 1.29, 95% CI 1.10-1.51; *P*=.002) and MAD (AOR 1.32, 95% CI 1.13-1.55; *P*<.001) to their children, respectively, than their counterparts. Similarly, maternal status of receiving ≥4 ANC visits was significantly associated with 23%, 31%, and 34% higher likelihood of providing an MMF (AOR 1.23, 95% CI 1.05-1.45; *P*=.01), MDD (AOR 1.31, 95% CI 1.13-1.51; *P*<.001), and MAD (AOR 1.34, 95% CI 1.15-1.56; *P*<.001) to their children, respectively, compared to mothers who did not receive ≥4 ANC.

In all regression models, the SE of the odds ratio was <2, and the VIF was <3, indicating the absence of collinearity among the independent variables. The detailed regression models are provided in Multimedia Appendix 1. There were some observations (n=83) with missing values in covariates in the analyzed sample. Therefore, we also conducted the analysis after excluding observations with missing values. However, we did not find any significant difference in the direction and significance of the association between the analysis including observations with missing values in covariates and the complete case analysis.

## Discussion

### Principal Findings

Our findings demonstrate significant positive effects of maternal use of the CoC on providing an MDD and MAD to their children. Breaking down the effect of individual CoC components, the status of receiving ≥4 ANC visits exhibited a significant positive association with MMF, MDD, and MAD. However, we did not find any significant effect of the status of maternal use of CoC or its components on ISSSF.

Consistent with our findings, a previous study using the BDHS 2007 data also reported no significant association between ISSSF and the number of ANC and PNC visits [32]. Timely ISSSF is associated with exclusive breastfeeding because children who are not exclusively breastfed are likely to receive family foods before the age of 6 months, which results in disqualifying them from receiving timely ISSSF. In Bangladesh, the exclusive breastfeeding rate declined from 64% in 2017 to 53% in 2022; therefore, this might partly explain the statistically insignificant effect of maternal use of the CoC and its components on timely ISSSF. There is a scarcity of studies explicitly examining the association between maternal CoC use and timely ISSSF. Previous studies highlight that complementary feeding practices are primarily influenced by maternal knowledge, cultural beliefs, family practices, and household factors rather than formal health care service use [19,33]. Similarly, a study in rural Bangladesh revealed that only 19% of mothers knew the appropriate age for introducing complementary foods, and this knowledge was not significantly associated with their feeding practices [34]. This provides an insight that, although an increase in maternal CoC use indicates a better coverage of maternal health care services, it may not ensure better child feeding practices (eg, ISSSF) unless the maternal health care services are strengthened with targeted nutrition education and behavior change interventions. The analysis revealed that mothers who received ≥4 ANC visits and the full CoC had significantly higher odds of providing an MMF than their counterparts. This finding is consistent with those of a previous study conducted using the Bangladesh Integrated Household Survey, which reported a positive association between ≥4 ANC visits and MMF [35]. Indeed, ANC provides platforms for counseling on nutrition as well as the importance of child feeding frequency and dietary adequacy. MAD is a composite indicator of both MMF and MDD. In this study, we found that the status of using the full CoC and receiving ≥4 ANC visits had a significant positive association with MAD. Similar studies based on the Bangladesh Integrated Household Survey 2018‐2019 and from other South Asian countries have also reported a positive association between ≥4 ANC visits and MAD [28,35-37]. Four or more ANC visits facilitate positive complementary feeding practices such as MDD and MAD through sustained opportunities for nutritional counseling [38]. Mothers who received the full CoC package were 29% more likely to provide foods meeting the MDD for their children. Notably, the trend of MDD in Bangladesh remained relatively consistent over a 10-year period from 2004 to 2014 [20]. Looking into the individual effects of the components of the CoC, the status of receiving ≥4 ANC visits also exhibited a significant association with MDD status. A previous study identified that mothers who received ≥4 ANC visits were 31% more likely to give their babies minimum diversified foods than those who did not have any ANC visits [39]. No significant effect of delivery by SBAs and PNC within 48 hours of delivery was observed on MDD. This could be because these services are more focused on maternal and essential newborn care rather than on complementary feeding practices, which require targeted counseling and behavior change communication. A previous study reported significant associations between dietary diversity score and status of receiving ≥4 ANC visits, which aligns with our findings [40].

This study was conducted using 2 consecutive nationally representative cross-sectional surveys (BDHS 2017‐2018 and 2022) conducted after the National Nutrition Policy 2015 was adopted in Bangladesh. Therefore, the findings provide a valuable insight into how the benefits of nutrition mainstreaming to maternal and child health care platforms are reflected in complementary feeding practices. The exposure variables included both maternal use of the CoC and its components (ie, ≥4 ANC visits, delivery assisted by SBAs, and PNC within 48 hours of delivery), which provides a holistic overview of their association with recommended complementary feeding practices and which maternal health care component should be further strengthened with policy support to increase the benefits. Furthermore, adjustment of covariates increased the accuracy of the estimates. The surveys use validated measurements for the exposure and outcome variables, strengthening the internal validity. On the other hand, the cross-sectional design limits causal inference between maternal use of the CoC and complementary feeding practices. Key behavioral factors such as maternal nutritional knowledge and attitude, cultural norms, household food security, and intrahousehold food dynamics were not estimated in the survey and, therefore, not included in this analysis. Furthermore, the quality of ANC and PNC, especially nutrition counseling while providing these services, was not considered in the statistical models. These issues should be considered carefully when interpreting and using the findings.

### Conclusions

Maternal use of the CoC, particularly through receiving ≥4 ANC visits, appears to be a significant determinant of improved MDD and MAD among children aged 6 to 23 months in Bangladesh. Enhancing maternal knowledge and practices related to exclusive breastfeeding may help facilitate the practice of timely ISSSF. Expanding maternal use of the CoC through policy support and targeted interventions presents a valuable opportunity to fully leverage its positive impact on maternal complementary feeding practices in Bangladesh.

## Supplementary material

10.2196/76666 Multimedia Appendix 1 Details of the regression model outcomes.

10.2196/76666 Checklist 1 STROBE checklist.

## Abbreviations

ANC: antenatal care
AOR: adjusted odds ratio
BDHS: Bangladesh Demographic and Health Survey
CoC: continuum of care
ISSSF: introduction to solid, semisolid, and soft food
MAD: minimum acceptable diet
MDD: minimum dietary diversity
MMF: minimum meal frequency
PNC: postnatal care
SBA: skilled birth attendant
STROBE: Strengthening the Reporting of Observational Studies in Epidemiology
VIF: variance inflation factor
