# Supplementary material for: Effects of Maternal Use of the Continuum of Care on Complementary Feeding Practices in Bangladesh: Cross-Sectional Study
Source: JMIR Public Health Surveill. 2025 Oct 7;11:e76666. doi: 10.2196/76666 (PMC12505405; doi:10.2196/76666)
Supplement: Multimedia Appendix 1 [file publichealth-v11-e76666-s001.pdf]

Effects of maternal utilization of continuum of care on complementary feeding practices in Bangladesh: unveiling bottlenecks and opportunities

Supplementary file

Table of Contents

Items **Page Number**

Supplementary table 1: Unadjusted and adjusted association between maternal utilization of the continuum of care and ISSSF. 3

Supplementary table 2: Unadjusted and adjusted association between maternal utilization of ≥ 4 ANC visits and ISSSF. 5

Supplementary table 3: Unadjusted and adjusted association between maternal utilization of delivery by SBA and ISSSF. 8

Supplementary table 4: Unadjusted and adjusted association between maternal utilization of PNC within 48 hours and ISSSF. 10

Supplementary table 5: Unadjusted and adjusted association between maternal utilization of the continuum of care and MMF. 13

Supplementary table 6: Unadjusted and adjusted association between maternal utilization of ≥ 4 ANC visits and MMF. 15

Supplementary table 7: Unadjusted and adjusted association between maternal utilization of delivery by SBA and MMF. 18

Supplementary table 8: Unadjusted and adjusted association between maternal utilization of PNC within 48 hours and MMF. 20

Supplementary table 9: Unadjusted and adjusted association between maternal utilization of the continuum of care and MDD. 23

Supplementary table 10: Unadjusted and adjusted association between maternal utilization of ≥ 4 ANC visits and MDD. 25

Supplementary table 11: Unadjusted and adjusted association between maternal utilization of delivery by SBA and MDD. 28

Supplementary table 12: Unadjusted and adjusted association between maternal utilization of PNC within 48 hours and MDD. 30

Supplementary table 13: Unadjusted and adjusted association between maternal utilization of the continuum of care and MAD. 33
Supplementary table 14: Unadjusted and adjusted association between maternal utilization of ≥ 4 ANC visits and MAD. 35
Supplementary table 15: Unadjusted and adjusted association between maternal utilization of delivery by SBA and MAD. 38
Supplementary table 16: Unadjusted and adjusted association between maternal utilization of PNC within 48 hours and MAD. 40

Supplementary table 1: Unadjusted and adjusted association between maternal utilization of the continuum of care and ISSSF.

Independent variables	OR	95% CI	p-value	AOR[α]	95% CI	p-value
**CoC**						
No	Ref	-	-	Ref	-	-
Yes	1.82	1.15, 2.88	0.011	1.30	0.77, 2.20	0.326
**Age at delivery**						
<19 years	Ref	-	-	Ref	-	-
19-30 years	0.71	0.40, 1.24	0.225	0.86	0.44, 1.66	0.648
31-49 years	1.15	0.57, 2.33	0.703	1.44	0.56, 3.71	0.449
**Education level**						
No education	Ref	-	-	Ref	-	-
Primary	1.01	0.47, 2.18	0.975	1.05	0.44, 2.48	0.915
Secondary	1.25	0.61, 2.60	0.541	1.25	0.50, 3.13	0.636
Higher	2.54	1.03, 6.28	0.043	1.78	0.57, 5.54	0.322
**Husband's education**						
No education	Ref	-	-	Ref	-	-
Primary	1.04	0.61, 1.77	0.899	0.87	0.47, 1.60	0.650
Secondary	1.10	0.63, 1.90	0.746	0.86	0.44, 1.69	0.659
Higher	2.25	1.09, 4.64	0.029	1.09	0.44, 2.70	0.845
**Occupation status**						
Not working	Ref	-	-	Ref	-	-
Working	1.68	1.13, 2.49	0.010	1.56	1.01, 2.39	0.043
**Husband's occupation**						
Not working	Ref	-	-	Ref	-	-
Working	0.89	0.26, 3.00	0.847	1.11	0.25, 4.96	0.888
**Parity**						
1	Ref	-	-	Ref	-	-
2-3	0.74	0.49, 1.10	0.136	0.79	0.49, 1.28	0.336

>3	0.69	0.39, 1.24	0.214	0.78	0.33, 1.85	0.570
**Ever terminated pregnancy**						
No	Ref	-	-	Ref	-	-
Yes	1.11	0.68, 1.79	0.682	1.09	0.66, 1.78	0.742
**Desired pregnancy**						
Yes	Ref	-	-	Ref	-	-
No	0.90	0.57, 1.41	0.633	0.98	0.58, 1.66	0.950
**Wealth index**						
Poorest	Ref	-	-	Ref	-	-
Poorer	1.69	0.95, 3.01	0.075	1.63	0.88, 3.03	0.121
Middle	1.98	1.12, 3.48	0.019	1.87	0.94, 3.70	0.074
Richer	1.20	0.71, 2.04	0.498	1.17	0.60, 2.27	0.648
Richest	2.09	1.11, 3.92	0.022	1.60	0.65, 3.99	0.308
**Media exposure**						
No	Ref	-	-	Ref	-	-
Yes	1.27	0.87, 1.87	0.219	0.93	0.60, 1.45	0.745
**Accessing healthcare is a big problem**						
No	Ref	-	-	Ref	-	-
Yes	0.58	0.39, 0.86	0.007	0.65	0.43, 1.00	0.052
**Religion**						
Muslim	Ref	-	-	Ref	-	-
Others	1.14	0.61, 2.11	0.686	1.01	0.54, 1.89	0.963
**Residence**						
Urban	Ref	-	-	Ref	-	-
Rural	0.69	0.44, 1.09	0.114	0.65	0.36, 1.15	0.137
**Division**						
Dhaka	Ref	-	-	Ref	-	-
Barisal	1.31	0.65, 2.65	0.454	1.80	0.87, 3.72	0.115
Chittagong	0.50	0.27, 0.93	0.030	0.67	0.35, 1.29	0.232

Khulna	3.71	1.49, 9.21	0.005	3.96	1.51, 10.41	0.005
Mymensingh	2.00	0.96, 4.17	0.065	2.85	1.30, 6.25	0.009
Rajshahi	1.66	0.77, 3.56	0.192	1.93	0.86, 4.31	0.110
Rangpur	1.69	0.83, 3.46	0.150	2.21	1.04, 4.73	0.040
Sylhet	0.76	0.41, 1.42	0.393	1.26	0.63, 2.49	0.513
**Survey round**						
BDHS 2017-18	Ref	-	-	Ref	-	-
BDHS 2022	0.88	0.60, 1.29	0.509	0.87	0.57, 1.34	0.535

$^{\alpha}$Adjusted for Primary Sampling Unit (PSU), sampling strata, sampling weight

Supplementary table 2: Unadjusted and adjusted association between maternal utilization of ≥ 4 ANC visits and ISSSF.

Independent variables	OR	95% CI	p-value	AOR$^{\alpha}$	95% CI	p-value
**≥ 4 ANC**						
No	Ref	-	–	Ref	-	-
Yes	1.40	0.94, 2.09	0.101	1.00	0.64, 1.57	0.984
**Age at delivery**						
<19 years	Ref	-	-	Ref	-	-
19-30 years	0.71	0.40, 1.24	0.225	0.85	0.44, 1.65	0.632
31-49 years	1.15	0.57, 2.33	0.703	1.45	0.56, 3.72	0.440
**Education level**						
No education	Ref	-	-	Ref	-	-
Primary	1.01	0.47, 2.18	0.975	1.03	0.43, 2.45	0.946
Secondary	1.25	0.61, 2.60	0.541	1.26	0.50, 3.15	0.625
Higher	2.54	1.03, 6.28	0.043	1.84	0.59, 5.71	0.293

**Husband's education**						
No education	Ref	-	-	Ref	-	-
Primary	1.04	0.61, 1.77	0.899	0.87	0.47, 1.61	0.661
Secondary	1.10	0.63, 1.90	0.746	0.87	0.44, 1.69	0.672
Higher	2.25	1.09, 4.64	0.029	1.17	0.48, 2.88	0.729
**Occupation status**						
Not working	Ref	-	-	Ref	-	-
Working	1.68	1.13, 2.49	0.010	1.55	1.01, 2.37	0.046
**Husband's occupation**						
Not working	Ref	-	-	Ref	-	-
Working	0.89	0.26, 3.00	0.847	1.04	0.23, 4.67	0.995
**Parity**						
1	Ref	-	-	Ref	-	-
2-3	0.74	0.49, 1.10	0.136	0.79	0.49, 1.26	0.321
>3	0.69	0.39, 1.24	0.214	0.77	0.32, 1.84	0.560
**Ever terminated pregnancy**						
No	Ref	-	-	Ref	-	-
Yes	1.11	0.68, 1.79	0.682	1.12	0.68, 1.83	0.656
**Desired pregnancy**						
Yes	Ref	-	-	Ref	-	-
No	0.90	0.57, 1.41	0.633	0.98	0.58, 1.64	0.935
**Wealth index**						
Poorest	Ref	-	-	Ref	-	-
Poorer	1.69	0.95, 3.01	0.075	1.65	0.89, 3.05	0.112
Middle	1.98	1.12, 3.48	0.019	1.91	0.95, 3.83	0.068
Richer	1.20	0.71, 2.04	0.498	1.20	0.62, 2.34	0.583
Richest	2.09	1.11, 3.92	0.022	1.67	0.68, 4.11	0.265

**Media exposure**						
No	Ref	-	-	Ref	-	-
Yes	1.27	0.87, 1.87	0.219	0.96	0.61, 1.50	0.844
**Accessing healthcare is a big problem**						
No	Ref	-	-	Ref	-	-
Yes	0.58	0.39, 0.86	0.007	0.64	0.42, 0.97	0.036
**Religion**						
Muslim	Ref	-	-	Ref	-	-
Others	1.14	0.61, 2.11	0.686	1.02	0.54, 1.93	0.951
**Residence**						
Urban	Ref	-	-	Ref	-	-
Rural	0.69	0.44, 1.09	0.114	0.64	0.36, 1.13	0.123
**Division**						
Dhaka	Ref	-	-	Ref	-	-
Barisal	1.31	0.65, 2.65	0.454	1.82	0.88, 3.77	0.106
Chittagong	0.50	0.27, 0.93	0.030	0.68	0.35, 1.30	0.244
Khulna	3.71	1.49, 9.21	0.005	4.05	1.54, 10.66	0.005
Mymensingh	2.00	0.96, 4.17	0.065	2.87	1.30, 6.32	0.009
Rajshahi	1.66	0.77, 3.56	0.192	1.93	0.85, 4.34	0.114
Rangpur	1.69	0.83, 3.46	0.150	2.22	1.05, 4.71	0.038
Sylhet	0.76	0.41, 1.42	0.393	1.27	0.64, 2.53	0.489
**Survey round**						
BDHS 2017-18	Ref	-	-	Ref	-	-
BDHS 2022	0.88	0.60, 1.29	0.509	0.87	0.57, 1.33	0.513

$^{\alpha}$Adjusted for Primary Sampling Unit (PSU), sampling strata, sampling weight.

Supplementary table 3: Unadjusted and adjusted association between maternal utilization of delivery by SBA and ISSSF.

Independent variables	OR	95% CI	p-value	AOR[α]	95% CI	p-value
**Delivery by SBA**						
No	Ref	-	–	Ref	-	-
Yes	1.32	0.92, 1.91	0.132	0.98	0.63, 1.52	0.919
**Age at delivery**						
<19 years	Ref	-	-	Ref	-	-
19-30 years	0.71	0.40, 1.24	0.225	0.85	0.44, 1.64	0.630
31-49 years	1.15	0.57, 2.33	0.703	1.45	0.56, 3.75	0.441
**Education level**						
No education	Ref	-	-	Ref	-	-
Primary	1.01	0.47, 2.18	0.975	1.03	0.44, 2.44	0.944
Secondary	1.25	0.61, 2.60	0.541	1.26	0.50, 3.17	0.620
Higher	2.54	1.03, 6.28	0.043	1.85	0.59, 5.77	0.289
**Husband's education**						
No education	Ref	-	-	Ref	-	-
Primary	1.04	0.61, 1.77	0.899	0.87	0.47, 1.61	0.661
Secondary	1.10	0.63, 1.90	0.746	0.87	0.44, 1.70	0.678
Higher	2.25	1.09, 4.64	0.029	1.18	0.47, 2.92	0.725
**Occupation status**						
Not working	Ref	-	-	Ref	-	-
Working	1.68	1.13, 2.49	0.010	1.54	1.00, 2.37	0.048

**Husband's occupation**						
Not working	Ref	-	-	Ref	-	-
Working	0.89	0.26, 3.00	0.847	1.04	0.23, 4.67	0.958
**Parity**						
1	Ref	-	-	Ref	-	-
2-3	0.74	0.49, 1.10	0.136	0.79	0.49, 1.26	0.321
>3	0.69	0.39, 1.24	0.214	0.77	0.32, 1.83	0.554
**Ever terminated pregnancy**						
No	Ref	-	-	Ref	-	-
Yes	1.11	0.68, 1.79	0.682	1.12	0.68, 1.84	0.650
**Desired pregnancy**						
Yes	Ref	-	-	Ref	-	-
No	0.90	0.57, 1.41	0.633	0.98	0.58, 1.64	0.935
**Wealth index**						
Poorest	Ref	-	-	Ref	-	-
Poorer	1.69	0.95, 3.01	0.075	1.65	0.90, 3.04	0.107
Middle	1.98	1.12, 3.48	0.019	1.92	0.96, 3.80	0.063
Richer	1.20	0.71, 2.04	0.498	1.21	0.62, 2.35	0.573
Richest	2.09	1.11, 3.92	0.022	1.68	0.68, 4.14	0.261
**Media exposure**						
No	Ref	-	-	Ref	-	-
Yes	1.27	0.87, 1.87	0.219	0.96	0.61, 1.50	0.853
**Accessing healthcare is a big problem**						
No	Ref	-	-	Ref	-	-
Yes	0.58	0.39, 0.86	0.007	0.63	0.41, 0.97	0.035
**Religion**						
Muslim	Ref	-	-	Ref	-	-
Others	1.14	0.61, 2.11	0.686	1.02	0.54, 1.93	0.943
**Residence**						
Urban	Ref	-	-	Ref	-	-
Rural	0.69	0.44, 1.09	0.114	0.63	0.36, 1.13	0.122
**Division**						
Dhaka	Ref	-	-	Ref	-	-
Barisal	1.31	0.65, 2.65	0.454	1.82	0.88, 3.78	0.105
Chittagong	0.50	0.27, 0.93	0.030	0.68	0.35, 1.30	0.242

Khulna	3.71	1.49, 9.21	0.005	4.06	1.54, 10.69	0.005
Mymensingh	2.00	0.96, 4.17	0.065	2.87	1.30, 6.33	0.009
Rajshahi	1.66	0.77, 3.56	0.192	1.92	0.85, 4.34	0.114
Rangpur	1.69	0.83, 3.46	0.150	2.22	1.04, 4.72	0.038
Sylhet	0.76	0.41, 1.42	0.393	1.27	0.64, 2.53	0.494
**Survey round**						
BDHS 2017-18	Ref	-	-	Ref	-	-
BDHS 2022	0.88	0.60, 1.29	0.509	0.87	0.57, 1.33	0.525

[α]Adjusted for Primary Sampling Unit (PSU), sampling strata, sampling weight.

Supplementary table 4: Unadjusted and adjusted association between maternal utilization of PNC within 48 hours and ISSSF.

Independent variables	OR	95% CI	p-value	AOR[α]	95% CI	p-value
**PNC within 48 hours**						
No	Ref	-	–	Ref	-	-
Yes	1.65	1.15, 2.37	0.007	1.27	0.83, 1.93	0.267
**Age at delivery**						
<19 years	Ref	-	-	Ref	-	-
19-30 years	0.71	0.40, 1.24	0.225	0.87	0.45, 1.68	0.673
31-49 years	1.15	0.57, 2.33	0.703	1.43	0.56, 3.67	0.455
**Maternal education**						
No education	Ref	-	-	Ref	-	-
Primary	1.01	0.47, 2.18	0.975	1.02	0.43, 2.40	0.968
Secondary	1.25	0.61, 2.60	0.541	1.22	0.49, 3.05	0.669
Higher	2.54	1.03, 6.28	0.043	1.74	0.56, 5.43	0.339
**Husband’s education**						

No education	Ref	-	-	Ref	-	-
Primary	1.04	0.61, 1.77	0.899	0.87	0.47, 1.61	0.664
Secondary	1.10	0.63, 1.90	0.746	0.85	0.43, 1.67	0.638
Higher	2.25	1.09, 4.64	0.029	1.11	0.44, 2.77	0.825
**Maternal occupation**						
Not working	Ref	-	-	Ref	-	-
Working	1.68	1.13, 2.49	0.010	1.56	1.02, 2.39	0.042
**Husband's occupation**						
Not working	Ref	-	-	Ref	-	-
Working	0.89	0.26, 3.00	0.847	1.09	0.25, 4.76	0.908
**Parity**						
1	Ref	-	-	Ref	-	-
2-3	0.74	0.49, 1.10	0.136	0.80	0.49, 1.29	0.354
>3	0.69	0.39, 1.24	0.214	0.79	0.33, 1.88	0.592
**Ever terminated pregnancy**						
No	Ref	-	-	Ref	-	-
Yes	1.11	0.68, 1.79	0.682	1.11	0.68, 1.81	0.681
**Desired pregnancy**						
Yes	Ref	-	-	Ref	-	-
No	0.90	0.57, 1.41	0.633	0.98	0.58, 1.64	0.928
**Wealth index**						
Poorest	Ref	-	-	Ref	-	-
Poorer	1.69	0.95, 3.01	0.075	1.61	0.87, 2.98	0.131
Middle	1.98	1.12, 3.48	0.019	1.88	0.95, 3.72	0.069
Richer	1.20	0.71, 2.04	0.498	1.15	0.59, 2.22	0.685
Richest	2.09	1.11, 3.92	0.022	1.62	0.65, 4.02	0.298
**Media exposure**						
No	Ref	-	-	Ref	-	-
Yes	1.27	0.87, 1.87	0.219	0.93	0.60, 1.45	0.752

**Accessing healthcare is a big problem**						
No	Ref	-	-	Ref	-	-
Yes	0.58	0.39, 0.86	0.007	0.65	0.43, 0.99	0.046
**Religion**						
Muslim	Ref	-	-	Ref	-	-
Others	1.14	0.61, 2.11	0.686	1.01	0.54, 1.89	0.981
**Residence**						
Urban	Ref	-	-	Ref	-	-
Rural	0.69	0.44, 1.09	0.114	0.65	0.36, 1.16	0.143
**Division**						
Dhaka	Ref	-	-	Ref	-	-
Barisal	1.31	0.65, 2.65	0.054	1.82	0.88, 3.79	0.107
Chittagong	0.50	0.27, 0.93	0.030	0.67	0.35, 1.29	0.230
Khulna	3.71	1.49, 9.21	0.005	3.93	1.50, 10.32	0.006
Mymensingh	2.00	0.96, 4.17	0.065	2.84	1.29, 6.24	0.010
Rajshahi	1.66	0.77, 3.56	0.192	1.91	0.85, 4.29	0.116
Rangpur	1.69	0.83, 3.46	0.150	2.21	1.03, 4.70	0.041
Sylhet	0.76	0.41, 1.42	0.393	1.28	0.64, 2.55	0.481
**Survey round**						
BDHS 2017-18	Ref	-	-	Ref	-	-
BDHS 2022	0.88	0.60, 1.29	0.509	0.86	0.56, 1.32	0.486

[α]Adjusted for Primary Sampling Unit (PSU), sampling strata, sampling weight.

Supplementary table 5: Unadjusted and adjusted association between maternal utilization of the continuum of care and MMF.

Independent variables	OR	95% CI	p-value	AOR[α]	95% CI	p-value
**CoC**						
No	Ref	-	–	Ref	-	-
Yes	1.53	1.28, 1.82	0.000	1.13	0.94, 1.37	0.202
**Age at delivery**						
<19 years	Ref	-	-	Ref	-	-
19-30 years	0.87	0.71, 1.06	0.168	0.95	0.75, 1.21	0.696
31-49 years	1.06	0.82, 1.36	0.669	1.18	0.86, 1.62	0.316
**Education level**						
No education	Ref	-	-	Ref	-	-
Primary	1.21	0.89, 1.66	0.223	1.19	0.84, 1.69	0.318
Secondary	1.32	0.97, 1.78	0.074	1.26	0.88, 1.80	0.209
Higher	1.97	1.40, 2.79	0.000	1.39	0.91, 2.14	0.132
**Husband's education**						
No education	Ref	-	-	Ref	-	-
Primary	1.42	1.14, 1.75	0.001	1.24	0.98, 1.57	0.070
Secondary	1.34	1.07, 1.67	0.009	1.14	0.89, 1.47	0.293
Higher	2.18	1.66, 2.85	0.000	1.56	1.12, 2.18	0.009
**Occupation status**						
Not working	Ref	-	-	Ref	-	-
Working	1.78	1.50, 2.11	0.000	1.60	1.33, 1.92	0.000
**Husband's occupation**						
Not working	Ref	-	-	Ref	-	-
Working	1.47	0.89, 2.42	0.129	1.51	0.85, 2.66	0.156

**Parity**						
1	Ref	-	-	Ref	-	-
2-3	0.84	0.72, 0.97	0.020	0.83	0.69, 1.01	0.058
>3	0.89	0.70, 1.14	0.359	0.95	0.69, 1.32	0.766
**Ever terminated pregnancy**						
No	Ref	-	-	Ref	-	-
Yes	1.12	0.93, 1.36	0.233	1.10	0.89, 1.35	0.367
**Desired pregnancy**						
Yes	Ref	-	-	Ref	-	-
No	1.11	0.93, 1.33	0.263	1.04	085, 1.26	0.700
**Wealth index**						
Poorest	Ref	-	-	Ref	-	-
Poorer	1.13	0.92, 1.39	0.231	1.07	0.86, 1.34	0.556
Middle	1.23	0.98, 1.54	0.073	1.09	0.84, 1.40	0.528
Richer	1.27	1.01, 1.59	0.041	1.00	0.76, 1.30	0.982
Richest	1.66	1.30, 2.12	0.000	1.11	0.81, 1.53	0.522
**Media exposure**						
No	Ref	-	-	Ref	-	-
Yes	1.55	1.34, 1.80	0.000	1.20	1.01, 1.42	0.035
**Accessing healthcare is a big problem**						
No	Ref	-	-	Ref	-	-
Yes	0.68	0.58, 0.79	0.000	0.76	0.65, 0.89	0.001
**Religion**						
Muslim	Ref	-	-	Ref	-	-
Others	1.19	0.85, 1.67	0.299	1.10	0.80, 1.51	0.560
**Residence**						
Urban	Ref	-	-	Ref	-	-
Rural	0.77	0.64, 0.93	0.007	0.85	0.69, 1.05	0.127
**Division**						

Dhaka	Ref	-	-	Ref	-	-
Barisal	0.57	0.43, 0.75	0.000	0.66	0.50, 0.89	0.005
Chittagong	0.52	0.41, 0.67	0.000	0.63	0.49, 0.80	0.000
Khulna	1.72	1.24, 2.37	0.001	1.87	1.33, 2.65	0.000
Mymensingh	0.99	0.75, 1.30	0.921	1.21	0.91, 1.60	0.187
Rajshahi	0.87	0.65, 1.16	0.332	0.86	0.64, 1.16	0.315
Rangpur	1.17	0.87, 1.57	0.297	1.25	0.91, 1.70	0.164
Sylhet	0.68	0.51, 0.91	0.009	0.87	0.65, 1.16	0.342
**Survey round**						
BDHS 2017-18	Ref	-	-	Ref	-	-
BDHS 2022	0.46	0.39, 0.54	0.000	0.48	0.41, 0.56	0.000

[α]Adjusted for Primary Sampling Unit (PSU), sampling strata, sampling weight.

Supplementary table 6: Unadjusted and adjusted association between maternal utilization of ≥ 4 ANC visits and MMF.

Independent variables	OR	95% CI	p-value	AOR[α]	95% CI	p-value
**≥ 4 ANC**						
No	Ref	-	–	Ref	-	-
Yes	1.62	1.39, 1.89	0.000	1.23	1.05, 1.45	0.012
**Age at delivery**						
<19 years	Ref	-	-	Ref	-	-
19-30 years	0.87	0.71, 1.06	0.168	0.95	0.75, 1.20	0.672
31-49 years	1.06	0.82, 1.36	0.669	0.17	0.85, 1.61	0.335
**Education level**						
No education	Ref	-	-	Ref	-	-
Primary	1.21	0.89, 1.66	0.223	1.19	0.84, 1.68	0.325

Secondary	1.32	0.97, 1.78	0.074	1.24	0.87, 1.78	0.231
Higher	1.97	1.40, 2.79	0.000	1.37	0.89, 2.10	0.149
**Husband's education**						
No education	Ref	-	-	Ref	-	-
Primary	1.42	1.14, 1.75	0.001	1.24	0.99, 1.57	0.066
Secondary	1.34	1.07, 1.67	0.009	1.15	0.89, 1.47	0.285
Higher	2.18	1.66, 2.85	0.000	1.55	1.11, 2.17	0.010
**Occupation status**						
Not working	Ref	-	-	Ref	-	-
Working	1.78	1.50, 2.11	0.000	1.59	1.33, 1.91	0.000
**Husband's occupation**						
Not working	Ref	-	-	Ref	-	-
Working	1.47	0.89, 2.42	0.129	1.51	0.86, 2.66	0.152
**Parity**						
1	Ref	-	-	Ref	-	-
2-3	0.84	0.72, 0.97	0.020	0.83	0.69, 1.01	0.060
>3	0.89	0.70, 1.14	0.359	0.96	0.69, 1.34	0.816
**Ever terminated pregnancy**						
No	Ref	-	-	Ref	-	-
Yes	1.12	0.93, 1.36	0.233	1.09	0.89, 1.34	0.419
**Desired pregnancy**						
Yes	Ref	-	-	Ref	-	-
No	1.11	0.93, 1.33	0.263	1.04	0.86, 1.27	0.675
**Wealth index**						
Poorest	Ref	-	-	Ref	-	-
Poorer	1.13	0.92, 1.39	0.231	1.07	0.85, 1.33	0.569
Middle	1.23	0.98, 1.54	0.073	1.08	0.83, 1.39	0.568
Richer	1.27	1.01, 1.59	0.041	0.99	0.76, 1.29	0.935
Richest	1.66	1.30, 2.12	0.000	1.09	0.79, 1.50	0.594

**Media exposure**						
No	Ref	-	-	Ref	-	-
Yes	1.55	1.34, 1.80	0.000	1.19	1.00, 1.41	0.048
**Accessing healthcare is a big problem**						
No	Ref	-	-	Ref	-	-
Yes	0.68	0.58, 0.79	0.000	0.77	0.65, 0.90	0.001
**Religion**						
Muslim	Ref	-	-	Ref	-	-
Others	1.19	0.85, 1.67	0.299	1.09	0.79, 1.50	0.596
**Residence**						
Urban	Ref	-	-	Ref	-	-
Rural	0.77	0.64, 0.93	0.007	0.86	0.70, 1.06	0.148
**Division**						
Dhaka	Ref	-	-	Ref	-	-
Barisal	0.57	0.43, 0.75	0.000	0.67	0.50, 0.90	0.007
Chittagong	0.52	0.41, 0.67	0.000	0.63	0.49, 0.81	0.000
Khulna	1.72	1.24, 2.37	0.001	1.88	1.33, 2.65	0.000
Mymensingh	0.99	0.75, 1.30	0.921	1.19	0.90, 1.57	0.221
Rajshahi	0.87	0.65, 1.16	0.332	0.86	0.64, 1.16	0.332
Rangpur	1.17	0.87, 1.57	0.297	1.23	0.90, 1.68	0.184
Sylhet	0.68	0.51, 0.91	0.009	0.87	0.65, 1.17	0.350
**Survey round**						
BDHS 2017-18	Ref	-	-	Ref	-	-
BDHS 2022	0.46	0.39, 0.54	0.000	0.48	0.41, 0.56	0.000

$^{\alpha}$Adjusted for Primary Sampling Unit (PSU), sampling strata, sampling weight.

Supplementary table 7: Unadjusted and adjusted association between maternal utilization of delivery by SBA and MMF.

Independent variables	OR	95% CI	p-value	AOR[α]	95% CI	p-value
**Delivery by SBA**						
No	Ref	-	–	Ref	-	-
Yes	1.06	0.91, 1.24	0.432	0.94	0.80, 1.11	0.489
**Age at delivery**						
<19 years	Ref	-	-	Ref	-	-
19-30 years	0.87	0.71, 1.06	0.168	0.95	0.75, 1.21	0.690
31-49 years	1.06	0.82, 1.36	0.669	1.18	0.86, 1.63	0.305
**Education level**						
No education	Ref	-	-	Ref	-	-
Primary	1.21	0.89, 1.66	0.223	1.20	0.84, 1.70	0.311
Secondary	1.32	0.97, 1.78	0.074	1.28	0.89, 1.84	0.176
Higher	1.97	1.40, 2.79	0.000	1.44	0.94, 2.22	0.096
**Husband's education**						
No education	Ref	-	-	Ref	-	-
Primary	1.42	1.14, 1.75	0.001	1.24	0.98, 1.56	0.070
Secondary	1.34	1.07, 1.67	0.009	1.15	0.90, 1.48	0.267
Higher	2.18	1.66, 2.85	0.000	1.60	1.14, 2.24	0.006
**Occupation status**						
Not working	Ref	-	-	Ref	-	-
Working	1.78	1.50, 2.11	0.000	1.59	1.32, 1.91	0.000
**Husband's occupation**						
Not working	Ref	-	-	Ref	-	-
Working	1.47	0.89, 2.42	0.129	1.51	0.85, 2.67	0.157
**Parity**						
1	Ref	-	-	Ref	-	-
2-3	0.84	0.72, 0.97	0.020	0.82	0.68, 1.00	0.047

>3	0.89	0.70, 1.14	0.359	0.94	0.67, 1.30	0.693
**Ever terminated pregnancy**						
No	Ref	-	-	Ref	-	-
Yes	1.12	0.93, 1.36	0.233	1.11	0.91, 1.37	0.303
**Desired pregnancy**						
Yes	Ref	-	-	Ref	-	-
No	1.11	0.93, 1.33	0.263	1.03	0.85, 1.26	0.751
**Wealth index**						
Poorest	Ref	-	-	Ref	-	-
Poorer	1.13	0.92, 1.39	0.231	1.07	0.86, 1.34	0.524
Middle	1.23	0.98, 1.54	0.073	1.10	0.85, 1.42	0.467
Richer	1.27	1.01, 1.59	0.041	1.02	0.78, 1.33	0.884
Richest	1.66	1.30, 2.12	0.000	1.15	0.84, 1.59	0.379
**Media exposure**						
No	Ref	-	-	Ref	-	-
Yes	1.55	1.34, 1.80	0.000	1.22	1.03, 1.44	0.024
**Accessing healthcare is a big problem**						
No	Ref	-	-	Ref	-	-
Yes	0.68	0.58, 0.79	0.000	0.75	0.64, 0.89	0.001
**Religion**						
Muslim	Ref	-	-	Ref	-	-
Others	1.19	0.85, 1.67	0.299	1.12	0.82, 1.54	0.481
**Residence**						
Urban	Ref	-	-	Ref	-	-
Rural	0.77	0.64, 0.93	0.007	0.84	0.69, 1.04	0.104
**Division**						
Dhaka	Ref	-	-	Ref	-	-
Barisal	0.57	0.43, 0.75	0.000	0.66	0.50, 0.89	0.005

Chittagong	0.52	0.41, 0.67	0.000	0.62	0.49, 0.80	0.000
Khulna	1.72	1.24, 2.37	0.001	1.90	1.35, 2.68	0.000
Mymensingh	0.99	0.75, 1.30	0.921	1.21	0.91, 1.60	0.188
Rajshahi	0.87	0.65, 1.16	0.332	0.86	0.64, 1.16	0.320
Rangpur	1.17	0.87, 1.57	0.297	1.25	0.92, 1.70	0.157
Sylhet	0.68	0.51, 0.91	0.009	0.87	0.65, 1.16	0.341
**Survey round**						
BDHS 2017-18	Ref	-	-	Ref	-	-
BDHS 2022	0.46	0.39, 0.54	0.000	0.48	0.41, 0.56	0.000

[α]Adjusted for Primary Sampling Unit (PSU), sampling strata, sampling weight.

Supplementary table 8: Unadjusted and adjusted association between maternal utilization of PNC within 48 hours and MMF.

Independent variables	OR	95% CI	p-value	AOR[α]	95% CI	p-value
**PNC within 48 hours**						
No	Ref	-	–	Ref	-	-
Yes	1.28	1.10, 1.48	0.002	1.05	0.89, 1.24	0.545
**Age at delivery**						
<19 years	Ref	-	-	Ref	-	-
19-30 years	0.87	0.71, 1.06	0.168	0.95	0.75, 1.20	0.677
31-49 years	1.06	0.82, 1.36	0.669	1.18	0.85, 1.62	0.321
**Education status**						
No education	Ref	-	-	Ref	-	-
Primary	1.21	0.89, 1.66	0.223	1.19	0.84, 1.69	0.316
Secondary	1.32	0.97, 1.78	0.074	1.27	0.88, 1.81	0.198
Higher	1.97	1.40, 2.79	0.000	1.41	0.92, 2.17	0.115

**Husband's education**						
No education	Ref	-	-	Ref	-	-
Primary	1.42	1.14, 1.75	0.001	1.24	0.98, 1.56	0.074
Secondary	1.34	1.07, 1.67	0.009	1.14	0.89, 1.47	0.298
Higher	2.18	1.66, 2.85	0.000	1.57	1.12, 2.20	0.008
**Occupation status**						
Not working	Ref	-	-	Ref	-	-
Working	1.78	1.50, 2.11	0.000	1.60	1.33, 1.92	0.000
**Husband's occupation**						
Not working	Ref	-	-	Ref	-	-
Working	1.47	0.89, 2.42	0.129	1.50	0.85, 2.65	0.159
**Parity**						
1	Ref	-	-	Ref	-	-
2-3	0.84	0.72, 0.97	0.020	0.83	0.69, 1.01	0.059
>3	0.89	0.70, 1.14	0.359	0.95	0.69, 1.32	0.768
**Ever terminated pregnancy**						
No	Ref	-	-	Ref	-	-
Yes	1.12	0.93, 1.36	0.233	1.11	0.90, 1.36	0.336
**Desired pregnancy**						
Yes	Ref	-	-	Ref	-	-
No	1.11	0.93, 1.33	0.263	1.04	0.85, 1.26	0.718
**Wealth index**						
Poorest	Ref	-	-	Ref	-	-
Poorer	1.13	0.92, 1.39	0.231	1.07	0.85, 1.33	0.568
Middle	1.23	0.98, 1.54	0.073	1.09	0.84, 1.40	0.528
Richer	1.27	1.01, 1.59	0.041	1.00	0.77, 1.31	0.986
Richest	1.66	1.30, 2.12	0.000	1.13	0.82, 1.55	0.461
**Media exposure**						
No	Ref	-	-	Ref	-	-

Yes	1.55	1.34, 1.80	0.000	1.21	1.02, 1.43	0.030
**Accessing healthcare is a big problem**						
No	Ref	-	-	Ref	-	-
Yes	0.68	0.58, 0.79	0.000	0.76	0.65, 0.89	0.001
**Religion**						
Muslim	Ref	-	-	Ref	-	-
Others	1.19	0.85, 1.67	0.299	1.11	0.80, 1.52	0.537
**Residence**						
Urban	Ref	-	-	Ref	-	-
Rural	0.77	0.64, 0.93	0.007	0.85	0.69, 1.04	0.115
**Division**						
Dhaka	Ref	-	-	Ref	-	-
Barisal	0.57	0.43, 0.75	0.000	0.66	0.50, 0.88	0.005
Chittagong	0.52	0.41, 0.67	0.000	0.63	0.49, 0.80	0.000
Khulna	1.72	1.24, 2.37	0.001	1.87	1.32, 2.64	0.000
Mymensingh	0.99	0.75, 1.30	0.921	1.21	0.92, 1.61	0.178
Rajshahi	0.87	0.65, 1.16	0.332	0.86	0.64, 1.15	0.313
Rangpur	1.17	0.87, 1.57	0.297	1.25	0.92, 1.70	0.158
Sylhet	0.68	0.51, 0.91	0.009	0.87	0.65, 1.17	0.351
**Survey round**						
BDHS 2017-18	Ref	-	-	Ref	-	-
BDHS 2022	0.46	0.39, 0.54	0.000	0.47	0.40, 0.56	0.000

$^{\alpha}$Adjusted for Primary Sampling Unit (PSU), sampling strata, sampling weight.

Supplementary table 9: Unadjusted and adjusted association between maternal utilization of the continuum of care and MDD.

Independent variables	OR	95% CI	p-value	AOR[α]	95% CI	p-value
**CoC**						
No	Ref	-	–	Ref	-	-
Yes	1.94	1.67, 2.25	0.000	1.29	1.10, 1.51	0.002
**Age at delivery**						
<19 years	Ref	-	-	Ref	-	-
19-30 years	0.99	0.83, 1.19	0.915	0.99	0.80, 1.22	0.923
31-49 years	1.07	0.85, 1.33	0.566	1.14	0.86, 1.51	0.363
**Education level**						
No education	Ref	-	-	Ref	-	-
Primary	1.72	1.23, 2.40	0.001	1.50	1.05, 2.15	0.025
Secondary	2.56	1.85, 3.54	0.000	1.87	1.29, 2.70	0.001
Higher	5.04	3.54, 7.18	0.000	2.54	1.66, 3.88	0.000
**Husband's education**						
No education	Ref	-	-	Ref	-	-
Primary	1.33	1.06, 1.68	0.016	1.10	0.86, 1.42	0.437
Secondary	1.75	1.40, 2.19	0.000	1.16	0.90, 1.49	0.264
Higher	3.24	2.55, 4.12	0.000	1.42	1.06, 1.91	0.020
**Occupation status**						
Not working	Ref	-	-	Ref	-	-
Working	1.14	1.00, 1.32	0.059	1.27	1.09, 1.48	0.002
**Husband's occupation**						
Not working	Ref	-	-	Ref	-	-
Working	1.55	0.95, 2.53	0.080	2.27	1.17, 4.37	0.015
**Parity**						
1	Ref	-	-	Ref	-	-
2-3	0.83	0.73, 0.95	0.006	0.91	0.77, 1.07	0.244

>3	0.61	0.48, 0.77	0.000	0.89	0.67, 1.20	0.455
**Ever terminated pregnancy**						
No	Ref	-	-	Ref	-	-
Yes	1.09	0.92, 1.28	0.305	1.07	0.89, 1.28	0.467
**Desired pregnancy**						
Yes	Ref	-	-	Ref	-	-
No	0.96	0.81, 1.13	0.610	1.05	0.88, 1.25	0.607
**Wealth index**						
Poorest	Ref	-	-	Ref	-	-
Poorer	1.44	1.17, 1.77	0.001	1.31	1.06, 1.63	0.014
Middle	1.56	1.26, 1.94	0.000	1.26	0.99, 1.60	0.066
Richer	1.91	1.55, 2.35	0.000	1.42	1.12, 1.81	0.004
Richest	3.37	2.70, 4.21	0.000	2.04	1.53, 2.71	0.000
**Media exposure**						
No	Ref	-	-	Ref	-	-
Yes	1.73	1.50, 2.00	0.000	1.20	1.02, 1.40	0.028
**Accessing healthcare is a big problem**						
No	Ref	-	-	Ref	-	-
Yes	0.77	0.67, 0.89	0.000	0.98	0.85, 1.13	0.830
**Religion**						
Muslim	Ref	-	-	Ref	-	-
Others	0.97	0.73, 1.28	0.814	0.86	0.65, 1.14	0.299
**Residence**						
Urban	Ref	-	-	Ref	-	-
Rural	0.71	0.60, 0.84	0.000	0.98	0.83, 1.16	0.828
**Division**						
Dhaka	Ref	-	-	Ref	-	-
Barisal	0.64	0.49, 0.83	0.001	0.83	0.63, 1.10	0.196
Chittagong	0.69	0.54, 0.87	0.002	0.84	0.66, 1.07	0.163

Khulna	1.18	0.93, 1.51	0.178	1.29	1.00, 1.66	0.050
Mymensingh	0.97	0.74, 1.26	0.798	1.34	1.02, 1.77	0.038
Rajshahi	0.79	0.61, 1.03	0.083	0.89	0.68, 1.16	0.379
Rangpur	1.00	0.79, 1.27	0.997	1.27	0.97, 1.66	0.081
Sylhet	0.57	0.45, 0.73	0.000	0.84	0.65, 1.08	0.173
**Survey round**						
BDHS 2017-18	Ref	-	-	Ref	-	-
BDHS 2022	0.95	0.82, 1.09	0.472	0.99	0.86, 1.15	0.929

$^{\alpha}$Adjusted for Primary Sampling Unit (PSU), sampling strata, sampling weight.

Supplementary table 10: Unadjusted and adjusted association between maternal utilization of ≥ 4 ANC visits and MDD.

Independent variables	OR	95% CI	p-value	AOR$^{\alpha}$	95% CI	p-value
**≥ 4 ANC**						
No	Ref	-	–	Ref	-	-
Yes	1.87	1.63, 2.15	0.000	1.31	1.13, 1.51	0.000
**Age at delivery**						
<19 years	Ref	-	-	Ref	-	-
19-30 years	0.99	0.83, 1.19	0.915	0.98	0.80, 1.21	0.877
31-49 years	1.07	0.85, 1.33	0.566	1.13	0.85, 1.50	0.390
**Maternal education**						
No education	Ref	-	-	Ref	-	-
Primary	1.72	1.23, 2.40	0.001	1.50	1.05, 2.14	0.026
Secondary	2.56	1.85, 3.54	0.000	1.86	1.29, 2.69	0.001
Higher	5.04	3.54, 7.18	0.000	2.54	1.66, 3.89	0.000

**Husband's education**						
No education	Ref	-	-	Ref	-	-
Primary	1.33	1.06, 1.68	0.016	1.11	0.86, 1.42	0.429
Secondary	1.75	1.40, 2.19	0.000	1.16	0.90, 1.50	0.254
Higher	3.24	2.55, 4.12	0.000	1.43	1.06, 1.92	0.019
**Maternal occupation**						
Not working	Ref	-	-	Ref	-	-
Working	1.14	1.00, 1.32	0.059	1.26	1.08, 1.47	0.003
**Husband's occupation**						
Not working	Ref	-	-	Ref	-	-
Working	1.55	0.95, 2.53	0.080	2.29	1.19, 4.39	0.013
**Parity**						
1	Ref	-	-	Ref	-	-
2-3	0.83	0.73, 0.95	0.006	0.91	0.77, 1.07	0.243
>3	0.61	0.48, 0.77	0.000	0.90	0.67, 1.21	0.488
**Ever terminated pregnancy**						
No	Ref	-	-	Ref	-	-
Yes	1.09	0.92, 1.28	0.305	1.06	0.89, 1.27	0.492
**Desired pregnancy**						
Yes	Ref	-	-	Ref	-	-
No	0.96	0.81, 1.13	0.610	1.05	0.88, 1.25	0.597
**Wealth index**						
Poorest	Ref	-	-	Ref	-	-
Poorer	1.44	1.17, 1.77	0.001	1.31	1.05, 1.63	0.015
Middle	1.56	1.26, 1.94	0.000	1.25	0.98, 1.59	0.074
Richer	1.91	1.55, 2.35	0.000	1.42	1.12, 1.82	0.004
Richest	3.37	2.70, 4.21	0.000	2.04	1.53, 2.71	0.000
**Media exposure**						

No	Ref	-	-	Ref	-	-
Yes	1.73	1.50, 2.00	0.000	1.19	1.01, 1.39	0.037
**Accessing healthcare is a big problem**						
No	Ref	-	-	Ref	-	-
Yes	0.77	0.67, 0.89	0.000	0.99	0.86, 1.14	0.845
**Religion**						
Muslim	Ref	-	-	Ref	-	-
Others	0.97	0.73, 1.28	0.814	0.86	0.65, 1.14	0.302
**Residence**						
Urban	Ref	-	-	Ref	-	-
Rural	0.71	0.60, 0.84	0.000	0.98	0.83, 1.17	0.850
**Division**						
Dhaka	Ref	-	-	Ref	-	-
Barisal	0.64	0.49, 0.83	0.001	0.85	0.64, 1.12	0.245
Chittagong	0.69	0.54, 0.87	0.002	0.86	0.67, 1.09	0.202
Khulna	1.18	0.93, 1.51	0.178	1.30	1.01, 1.67	0.044
Mymensingh	0.97	0.74, 1.26	0.798	1.32	1.00, 1.75	0.050
Rajshahi	0.79	0.61, 1.03	0.083	0.89	0.68, 1.17	0.407
Rangpur	1.00	0.79, 1.27	0.997	1.25	0.96, 1.64	0.098
Sylhet	0.57	0.45, 0.73	0.000	0.84	0.65, 1.09	0.182
**Survey round**						
BDHS 2017-18	Ref	-	-	Ref	-	-
BDHS 2022	0.95	0.82, 1.09	0.472	0.99	0.86, 1.14	0.903

$^{\alpha}$Adjusted for Primary Sampling Unit (PSU), sampling strata, sampling weight.

Supplementary table 11: Unadjusted and adjusted association between maternal utilization of delivery by SBA and MDD.

Independent variables	OR	95% CI	p-value	AOR[α]	95% CI	p-value
**Delivery by SBA**						
No	Ref	-	–	Ref	-	-
Yes	1.53	1.33, 1.76	0.000	1.04	0.89, 1.22	0.621
**Age at delivery**						
<19 years	Ref	-	-	Ref	-	-
19-30 years	0.99	0.83, 1.19	0.915	0.98	0.80, 1.21	0.875
31-49 years	1.07	0.85, 1.33	0.566	1.13	0.86, 1.50	0.383
**Education level**						
No education	Ref	-	-	Ref	-	-
Primary	1.72	1.23, 2.40	0.001	1.51	1.06, 2.16	0.024
Secondary	2.56	1.85, 3.54	0.000	1.91	1.32, 2.76	0.001
Higher	5.04	3.54, 7.18	0.000	2.65	1.73, 4.05	0.000
**Husband's education**						
No education	Ref	-	-	Ref	-	-
Primary	1.33	1.06, 1.68	0.016	1.10	0.86, 1.41	0.455
Secondary	1.75	1.40, 2.19	0.000	1.16	0.90, 1.50	0.242
Higher	3.24	2.55, 4.12	0.000	1.46	1.09, 1.96	0.012
**Occupation status**						
Not working	Ref	-	-	Ref	-	-
Working	1.14	1.00, 1.32	0.059	1.27	1.09, 1.48	0.003
**Husband's occupation**						
Not working	Ref	-	-	Ref	-	-
Working	1.55	0.95, 2.53	0.080	2.28	1.19, 4.38	0.013
**Parity**						
1	Ref	-	-	Ref	-	-
2-3	0.83	0.73, 0.95	0.006	0.90	0.77, 1.07	0.235

>3	0.61	0.48, 0.77	0.000	0.89	0.66, 1.19	0.432
**Ever terminated pregnancy**						
No	Ref	-	-	Ref	-	-
Yes	1.09	0.92, 1.28	0.305	1.09	0.91, 1.30	0.363
**Desired pregnancy**						
Yes	Ref	-	-	Ref	-	-
No	0.96	0.81, 1.13	0.610	1.04	0.87, 1.24	0.668
**Wealth index**						
Poorest	Ref	-	-	Ref	-	-
Poorer	1.44	1.17, 1.77	0.001	1.32	1.06, 1.64	0.013
Middle	1.56	1.26, 1.94	0.000	1.26	0.99, 1.61	0.064
Richer	1.91	1.55, 2.35	0.000	1.45	1.14, 1.86	0.003
Richest	3.37	2.70, 4.21	0.000	2.13	1.60, 2.83	0.000
**Media exposure**						
No	Ref	-	-	Ref	-	-
Yes	1.73	1.50, 2.00	0.000	1.22	1.04, 1.43	0.016
**Accessing healthcare is a big problem**						
No	Ref	-	-	Ref	-	-
Yes	0.77	0.67, 0.89	0.000	0.97	0.84, 1.12	0.698
**Religion**						
Muslim	Ref	-	-	Ref	-	-
Others	0.97	0.73, 1.28	0.814	0.88	0.66, 1.16	0.360
**Residence**						
Urban	Ref	-	-	Ref	-	-
Rural	0.71	0.60, 0.84	0.000	0.97	0.81, 1.15	0.690
**Division**						
Dhaka	Ref	-	-	Ref	-	-
Barisal	0.64	0.49, 0.83	0.001	0.83	0.63, 1.10	0.192

Chittagong	0.69	0.54, 0.87	0.002	0.84	0.66, 1.07	0.164
Khulna	1.18	0.93, 1.51	0.178	1.30	1.01, 1.68	0.042
Mymensingh	0.97	0.74, 1.26	0.798	1.35	1.02, 1.78	0.034
Rajshahi	0.79	0.61, 1.03	0.083	0.89	0.68, 1.16	0.394
Rangpur	1.00	0.79, 1.27	0.997	1.28	0.98, 1.67	0.070
Sylhet	0.57	0.45, 0.73	0.000	0.84	0.65, 1.08	0.180
**Survey round**						
BDHS 2017-18	Ref	-	-	Ref	-	-
BDHS 2022	0.95	0.82, 1.09	0.472	0.97	0.84, 1.12	0.663

[α]Adjusted for Primary Sampling Unit (PSU), sampling strata, sampling weight.

Supplementary table 12: Unadjusted and adjusted association between maternal utilization of PNC within 48 hours and MDD.

Independent variables	OR	95% CI	p-value	AOR[α]	95% CI	p-value
**PNC within 48 hours**						
No	Ref	-	–	Ref	-	-
Yes	1.41	1.22, 1.61	0.000	0.99	0.85, 1.15	0.879
**Age at delivery**						
<19 years	Ref	-	-	Ref	-	-
19-30 years	0.99	0.83, 1.19	0.915	0.98	0.80, 1.21	0.886
31-49 years	1.07	0.85, 1.33	0.566	1.14	0.86, 1.50	0.372
**Education level**						
No education	Ref	-	-	Ref	-	-
Primary	1.72	1.23, 2.40	0.001	1.51	1.06, 2.16	0.024
Secondary	2.56	1.85, 3.54	0.000	1.92	1.33, 2.77	0.001
Higher	5.04	3.54, 7.18	0.000	2.67	1.75, 4.08	0.000

**Husband's education**						
No education	Ref	-	-	Ref	-	-
Primary	1.33	1.06, 1.68	0.016	1.10	0.86, 1.41	0.450
Secondary	1.75	1.40, 2.19	0.000	1.17	0.91, 1.50	0.232
Higher	3.24	2.55, 4.12	0.000	1.47	1.10, 1.98	0.010
**Occupation status**						
Not working	Ref	-	-	Ref	-	-
Working	1.14	1.00, 1.32	0.059	1.26	1.08, 1.47	0.003
**Husband's occupation**						
Not working	Ref	-	-	Ref	-	-
Working	1.55	0.95, 2.53	0.080	2.29	1.19, 4.39	0.013
**Parity**						
1	Ref	-	-	Ref	-	-
2-3	0.83	0.73, 0.95	0.006	0.90	0.76, 1.06	0.214
>3	0.61	0.48, 0.77	0.000	0.88	0.65, 1.19	0.401
**Ever terminated pregnancy**						
No	Ref	-	-	Ref	-	-
Yes	1.09	0.92, 1.28	0.305	1.09	0.91, 1.30	0.345
**Desired pregnancy**						
Yes	Ref	-	-	Ref	-	-
No	0.96	0.81, 1.13	0.610	1.04	0.87, 1.24	0.689
**Wealth index**						
Poorest	Ref	-	-	Ref	-	-
Poorer	1.44	1.17, 1.77	0.001	1.32	1.06, 1.64	0.012
Middle	1.56	1.26, 1.94	0.000	1.27	0.99, 1.62	0.057
Richer	1.91	1.55, 2.35	0.000	1.47	1.15, 1.87	0.002
Richest	3.37	2.70, 4.21	0.000	2.15	1.62, 2.86	0.000

**Media exposure**						
No	Ref	-	-	Ref	-	-
Yes	1.73	1.50, 2.00	0.000	1.22	1.04, 1.43	0.014
**Accessing healthcare is a big problem**						
No	Ref	-	-	Ref	-	-
Yes	0.77	0.67, 0.89	0.000	0.97	0.84, 1.12	0.676
**Religion**						
Muslim	Ref	-	-	Ref	-	-
Others	0.97	0.73, 1.28	0.814	0.88	0.67, 1.17	0.379
**Residence**						
Urban	Ref	-	-	Ref	-	-
Rural	0.71	0.60, 0.84	0.000	0.96	0.81, 1.14	0.666
**Division**						
Dhaka	Ref	-	-	Ref	-	-
Barisal	0.64	0.49, 0.83	0.001	0.83	0.64, 1.10	0.195
Chittagong	0.69	0.54, 0.87	0.002	0.84	0.66, 1.07	0.162
Khulna	1.18	0.93, 1.51	0.178	1.31	1.02, 1.69	0.038
Mymensingh	0.97	0.74, 1.26	0.798	1.34	1.02, 1.77	0.036
Rajshahi	0.79	0.61, 1.03	0.083	0.89	0.68, 1.16	0.397
Rangpur	1.00	0.79, 1.27	0.997	1.28	0.98, 1.67	0.070
Sylhet	0.57	0.45, 0.73	0.000	0.84	0.65, 1.08	0.175
**Survey round**						
BDHS 2017-18	Ref	-	-	Ref	-	-
BDHS 2022	0.95	0.82, 1.09	0.472	0.97	0.84, 1,12	0.710

$^{\alpha}$Adjusted for Primary Sampling Unit (PSU), sampling strata, sampling weight.

Supplementary table 13: Unadjusted and adjusted association between maternal utilization of the continuum of care and MAD.

Independent variables	OR	95% CI	p-value	AOR[α]	95% CI	p-value
**CoC**						
No	Ref	-	–	Ref	-	-
Yes	1.98	1.71, 2.30	0.000	1.32	1.13, 1.55	0.001
**Age at delivery**						
<19 years	Ref	-	-	Ref	-	-
19-30 years	0.96	0.80, 1.15	0.641	0.93	0.75, 1.16	0.539
31-49 years	1.09	0.87, 1.38	0.451	1.13	0.83, 1.52	0.442
**Education level**						
No education	Ref	-	-	Ref	-	-
Primary	1.86	1.32, 2.62	0.000	1.64	1.14, 2.37	0.008
Secondary	2.62	1.86, 3.70	0.000	2.00	1.37, 2.93	0.000
Higher	4.99	3.48, 7.17	0.000	2.62	1.71, 4.02	0.000
**Husband's education**						
No education	Ref	-	-	Ref	-	-
Primary	1.42	1.12, 1.81	0.004	1.16	0.90, 1.50	0.242
Secondary	1.68	1.32, 2.13	0.000	1.12	0.86, 1.45	0.395
Higher	3.22	2.51, 4.12	0.000	1.49	1.09, 2.02	0.011
**Occupation status**						
Not working	Ref	-	-	Ref	-	-
Working	1.29	1.11, 1.49	0.001	1.38	1.18, 1.61	0.000
**Husband's occupation**						
Not working	Ref	-	-	Ref	-	-
Working	1.91	1.13, 3.25	0.016	2.77	1.32, 5.83	0.007
**Parity**						
1	Ref	-	-	Ref	-	-
2-3	0.86	0.75, 0.99	0.032	0.95	0.80, 1.12	0.526

>3	0.68	0.53, 0.86	0.002	0.97	0.72, 1.31	0.836
**Ever terminated pregnancy**						
No	Ref	-	-	Ref	-	-
Yes	1.09	0.92, 1.28	0.333	1.04	0.87, 1.25	0.663
**Desired pregnancy**						
Yes	Ref	-	-	Ref	-	-
No	1.00	0.85, 1.19	0.960	1.06	0.88, 1.26	0.543
**Wealth index**						
Poorest	Ref	-	-	Ref	-	-
Poorer	1.46	1.18, 1.81	0.001	1.31	1.04, 1.64	0.020
Middle	1.57	1.25, 1.96	0.000	1.23	0.96, 1.58	0.102
Richer	1.90	1.54, 2.34	0.000	1.36	1.06, 1.74	0.014
Richest	3.15	2.52, 3.94	0.000	1.83	1.37, 2.45	0.000
**Media exposure**						
No	Ref	-	-	Ref	-	-
Yes	1.86	1.60, 2.16	0.000	1.30	1.10, 1.53	0.002
**Accessing healthcare is a big problem**						
No	Ref	-	-	Ref	-	-
Yes	0.76	0.66, 0.87	0.000	0.95	0.82, 1.10	0.521
**Religion**						
Muslim	Ref	-	-	Ref	-	-
Others	0.97	0.73, 1.29	0.845	0.86	0.64, 1.15	0.310
**Residence**						
Urban	Ref	-	-	Ref	-	-
Rural	0.72	0.61, 0.85	0.000	0.98	0.82, 1.17	0.814
**Division**						
Dhaka	Ref	-	-	Ref	-	-
Barisal	0.64	0.49, 0.84	0.001	0.82	0.62, 1.10	0.185

Chittagong	0.64	0.50, 0.83	0.001	0.80	0.62, 1.03	0.079
Khulna	1.24	0.97, 1.58	0.085	1.34	1.02, 1.74	0.032
Mymensingh	0.93	0.70, 1.22	0.598	1.26	0.94, 1.68	0.122
Rajshahi	0.82	0.63, 1.06	0.129	0.87	0.66, 1.14	0.302
Rangpur	0.96	0.75, 1.23	0.775	1.17	0.89, 1.55	0.262
Sylhet	0.55	0.43, 0.72	0.000	0.82	0.62, 1.07	0.136
**Survey round**						
BDHS 2017-18	Ref	-	-	Ref	-	-
BDHS 2022	0.77	0.66, 0.89	0.000	0.81	0.70, 0.94	0.006

[α]Adjusted for Primary Sampling Unit (PSU), sampling strata, sampling weight.

Supplementary table 14: Unadjusted and adjusted association between maternal utilization of ≥ 4 ANC visits and MAD.

Independent variables	OR	95% CI	p-value	AOR[α]	95% CI	p-value
**≥ 4 ANC**						
No	Ref	-	–	Ref	-	-
Yes	1.91	1.66, 2.20	0.000	1.34	1.15, 1.56	0.000
**Age at delivery**						
<19 years	Ref	-	-	Ref	-	-
19-30 years	0.96	0.80, 1.15	0.641	0.93	0.75, 1.15	0.499
31-49 years	1.09	0.87, 1.38	0.451	1.12	0.83, 1.51	0.473
**Education level**						
No education	Ref	-	-	Ref	-	-
Primary	1.86	1.32, 2.62	0.000	1.64	1.14, 2.36	0.008
Secondary	2.62	1.86, 3.70	0.000	1.99	1.36, 2.92	0.000
Higher	4.99	3.48, 7.17	0.000	2.63	1.71, 4.03	0.000

**Husband's education**						
No education	Ref	-	-	Ref	-	-
Primary	1.42	1.12, 1.81	0.004	1.16	0.91, 1.50	0.236
Secondary	1.68	1.32, 2.13	0.000	1.12	0.87, 1.45	0.380
Higher	3.22	2.51, 4.12	0.000	1.49	1.10, 2.02	0.010
**Occupation status**						
Not working	Ref	-	-	Ref	-	-
Working	1.29	1.11, 1.49	0.001	1.37	1.17, 1.59	0.000
**Husband's occupation**						
Not working	Ref	-	-	Ref	-	-
Working	1.91	1.13, 3.25	0.016	2.81	1.34, 5.92	0.006
**Parity**						
1	Ref	-	-	Ref	-	-
2-3	0.86	0.75, 0.99	0.032	0.95	0.80, 1.12	0.523
>3	0.68	0.53, 0.86	0.002	0.98	0.72, 1.32	0.878
**Ever terminated pregnancy**						
No	Ref	-	-	Ref	-	-
Yes	1.09	0.92, 1.28	0.333	1.04	0.86, 1.25	0.688
**Desired pregnancy**						
Yes	Ref	-	-	Ref	-	-
No	1.00	0.85, 1.19	0.960	1.06	0.88, 1.27	0.534
**Wealth index**						
Poorest	Ref	-	-	Ref	-	-
Poorer	1.46	1.18, 1.81	0.001	1.30	1.04, 1.63	0.023
Middle	1.57	1.25, 1.96	0.000	1.22	0.95, 1.57	0.115
Richer	1.90	1.54, 2.34	0.000	1.37	1.07, 1.75	0.013
Richest	3.15	2.52, 3.94	0.000	1.84	1.37, 2.46	0.000

**Media exposure**						
No	Ref	-	-	Ref	-	-
Yes	1.86	1.60, 2.16	0.000	1.28	1.09, 1.51	0.003
**Accessing healthcare is a big problem**						
No	Ref	-	-	Ref	-	-
Yes	0.76	0.66, 0.87	0.000	0.95	0.82, 1.10	0.528
**Religion**						
Muslim	Ref	-	-	Ref	-	-
Others	0.97	0.73, 1.29	0.845	0.86	0.64, 1.16	0.315
**Residence**						
Urban	Ref	-	-	Ref	-	-
Rural	0.72	0.61, 0.85	0.000	0.98	0.82, 1.17	0.830
**Division**						
Dhaka	Ref	-	-	Ref	-	-
Barisal	0.64	0.49, 0.84	0.001	0.84	0.63, 1.12	0.234
Chittagong	0.64	0.50, 0.83	0.001	0.81	0.63, 1.04	0.100
Khulna	1.24	0.97, 1.58	0.085	1.35	1.03, 1.75	0.027
Mymensingh	0.93	0.70, 1.22	0.598	1.23	0.92, 1.65	0.154
Rajshahi	0.82	0.63, 1.06	0.129	0.87	0.67, 1.15	0.327
Rangpur	0.96	0.75, 1.23	0.775	1.16	0.88, 1.53	0.305
Sylhet	0.55	0.43, 0.72	0.000	0.82	0.63, 1.07	0.143
**Survey round**						
BDHS 2017-18	Ref	-	-	Ref	-	-
BDHS 2022	0.77	0.66, 0.89	0.000	0.81	0.70, 0.94	0.005

$^{\alpha}$Adjusted for Primary Sampling Unit (PSU), sampling strata, sampling weight.

Supplementary table 15: Unadjusted and adjusted association between maternal utilization of delivery by SBA and MAD.

Independent variables	OR	95% CI	p-value	AOR[α]	95% CI	p-value
**Delivery by SBA**						
No	Ref	-	–	Ref	-	-
Yes	1.44	1.24, 1.67	0.000	1.03	0.87, 1.21	0.772
**Age at delivery**						
<19 years	Ref	-	-	Ref	-	-
19-30 years	0.96	0.80, 1.15	0.641	0.93	0.75, 1.15	0.500
31-49 years	1.09	0.87, 1.38	0.451	0.12	0.83, 1.51	0.462
**Education level**						
No education	Ref	-	-	Ref	-	-
Primary	1.86	1.32, 2.62	0.000	1.65	1.14, 2.39	0.008
Secondary	2.62	1.86, 3.70	0.000	2.06	1.40, 3.01	0.000
Higher	4.99	3.48, 7.17	0.000	2.76	1.80, 4.23	0.000
**Husband's education**						
No education	Ref	-	-	Ref	-	-
Primary	1.42	1.12, 1.81	0.004	1.16	0.90, 1.49	0.254
Secondary	1.68	1.32, 2.13	0.000	1.13	0.87, 1.46	0.357
Higher	3.22	2.51, 4.12	0.000	1.54	1.13, 2.08	0.006
**Occupation status**						
Not working	Ref	-	-	Ref	-	-
Working	1.29	1.11, 1.49	0.001	1.37	1.17, 1.60	0.000
**Husband's occupation**						
Not working	Ref	-	-	Ref	-	-
Working	1.91	1.13, 3.25	0.016	2.80	1.33, 5.87	0.007
**Parity**						
1	Ref	-	-	Ref	-	-
2-3	0.86	0.75, 0.99	0.032	0.94	0.79, 1.12	0.493

>3	0.68	0.53, 0.86	0.002	0.96	0.71, 1.30	0.785
**Ever terminated pregnancy**						
No	Ref	-	-	Ref	-	-
Yes	1.09	0.92, 1.28	0.333	1.06	0.88, 1.27	0.519
**Desired pregnancy**						
Yes	Ref	-	-	Ref	-	-
No	1.00	0.85, 1.19	0.960	1.05	0.88, 1.25	0.615
**Wealth index**						
Poorest	Ref	-	-	Ref	-	-
Poorer	1.46	1.18, 1.81	0.001	1.31	1.05, 1.64	0.018
Middle	1.57	1.25, 1.96	0.000	1.24	0.96, 1.59	0.096
Richer	1.90	1.54, 2.34	0.000	1.40	1.09, 1.80	0.008
Richest	3.15	2.52, 3.94	0.000	1.94	1.45, 2.59	0.000
**Media exposure**						
No	Ref	-	-	Ref	-	-
Yes	1.86	1.60, 2.16	0.000	1.32	1.12, 1.55	0.001
**Accessing healthcare is a big problem**						
No	Ref	-	-	Ref	-	-
Yes	0.76	0.66, 0.87	0.000	0.94	0.81, 1.09	0.402
**Religion**						
Muslim	Ref	-	-	Ref	-	-
Others	0.97	0.73, 1.29	0.845	0.88	0.65, 1.18	0.387
**Residence**						
Urban	Ref	-	-	Ref	-	-
Rural	0.72	0.61, 0.85	0.000	0.96	0.80, 1.15	0.652
**Division**						
Dhaka	Ref	-	-	Ref	-	-
Barisal	0.64	0.49, 0.84	0.001	0.82	0.62, 1.09	0.181

Chittagong	0.64	0.50, 0.83	0.001	0.80	0.62, 1.02	0.077
Khulna	1.24	0.97, 1.58	0.085	1.35	1.04, 1.76	0.025
Mymensingh	0.93	0.70, 1.22	0.598	1.26	0.94, 1.68	0.116
Rajshahi	0.82	0.63, 1.06	0.129	0.87	0.66, 1.14	0.318
Rangpur	0.96	0.75, 1.23	0.775	1.19	0.90, 1.57	0.232
Sylhet	0.55	0.43, 0.72	0.000	0.82	0.63, 1.07	0.139
**Survey round**						
BDHS 2017-18	Ref	-	-	Ref	-	-
BDHS 2022	0.77	0.66, 0.89	0.000	0.79	0.68, 0.92	0.002

$^{\alpha}$Adjusted for Primary Sampling Unit (PSU), sampling strata, sampling weight.

Supplementary table 16: Unadjusted and adjusted association between maternal utilization of PNC within 48 hours and MAD.

Independent variables	OR	95% CI	p-value	AOR$^{\alpha}$	95% CI	p-value
**PNC within 48 hours**						
No	Ref	-	–	Ref	-	-
Yes	1.44	1.25, 1.66	0.000	1.03	0.88, 1.20	0.720
**Age at delivery**						
<19 years	Ref	-	-	Ref	-	-
19-30 years	0.96	0.80, 1.15	0.641	0.93	0.75, 1.15	0.501
31-49 years	1.09	0.87, 1.38	0.451	1.12	0.83, 1.51	0.459
**Education level**						
No education	Ref	-	-	Ref	-	-
Primary	1.86	1.32, 2.62	0.000	1.65	1.14, 2.39	0.008
Secondary	2.62	1.86, 3.70	0.000	2.06	1.40, 3.01	0.000

Higher	4.99	3.48, 7.17	0.000	2.76	1.80, 4.23	0.000
**Husband's education**						
No education	Ref	-	-	Ref	-	-
Primary	1.42	1.12, 1.81	0.004	1.16	0.90, 1.49	0.257
Secondary	1.68	1.32, 2.13	0.000	1.13	0.87, 1.46	0.361
Higher	3.22	2.51, 4.12	0.000	1.53	1.13, 2.08	0.006
**Occupation status**						
Not working	Ref	-	-	Ref	-	-
Working	1.29	1.11, 1.49	0.001	1.37	1.17, 1.60	0.000
**Husband's occupation**						
Not working	Ref	-	-	Ref	-	-
Working	1.91	1.13, 3.25	0.016	2.79	1.33, 5.85	0.007
**Parity**						
1	Ref	-	-	Ref	-	-
2-3	0.86	0.75, 0.99	0.032	0.94	0.79, 1.12	0.489
>3	0.68	0.53, 0.86	0.002	0.96	0.71, 1.30	0.779
**Ever terminated pregnancy**						
No	Ref	-	-	Ref	-	-
Yes	1.09	0.92, 1.28	0.333	1.06	0.89, 1.27	0.516
**Desired pregnancy**						
Yes	Ref	-	-	Ref	-	-
No	1.00	0.85, 1.19	0.960	1.05	0.88, 1.25	0.619
**Wealth index**						
Poorest	Ref	-	-	Ref	-	-
Poorer	1.46	1.18, 1.81	0.001	1.31	1.05, 1.64	0.019
Middle	1.57	1.25, 1.96	0.000	1.24	0.96, 1.59	0.094
Richer	1.90	1.54, 2.34	0.000	1.40	1.09, 1.79	0.008
Richest	3.15	2.52, 3.94	0.000	1.94	1.45, 2.59	0.000

**Media exposure**						
No	Ref	-	-	Ref	-	-
Yes	1.86	1.60, 2.16	0.000	1.32	1.12, 1.55	0.001
**Accessing healthcare is a big problem**						
No	Ref	-	-	Ref	-	-
Yes	0.76	0.66, 0.87	0.000	0.94	0.81, 1.09	0.401
**Religion**						
Muslim	Ref	-	-	Ref	-	-
Others	0.97	0.73, 1.29	0.845	0.88	0.65, 1.18	0.386
**Residence**						
Urban	Ref	-	-	Ref	-	-
Rural	0.72	0.61, 0.85	0.000	0.96	0.80, 1.15	0.650
**Division**						
Dhaka	Ref	-	-	Ref	-	-
Barisal	0.64	0.49, 0.84	0.001	0.82	0.62, 1.09	0.181
Chittagong	0.64	0.50, 0.83	0.001	0.80	0.62, 1.02	0.076
Khulna	1.24	0.97, 1.58	0.085	1.35	1.04, 1.76	0.026
Mymensingh	0.93	0.70, 1.22	0.598	1.26	0.94, 1.68	0.117
Rajshahi	0.82	0.63, 1.06	0.129	0.87	0.66, 1.14	0.315
Rangpur	0.96	0.75, 1.23	0.775	1.19	0.90, 1.57	0.232
Sylhet	0.55	0.43, 0.72	0.000	0.82	0.63, 1.07	0.139
**Survey round**						
BDHS 2017-18	Ref	-	-	Ref	-	-
BDHS 2022	0.77	0.66, 0.89	0.000	0.79	0.68, 0.92	0.002

$^{\alpha}$Adjusted for Primary Sampling Unit (PSU), sampling strata, sampling weight.
